# Biopolymers-Derived Materials for Supercapacitors: Recent Trends, Challenges, and Future Prospects

**DOI:** 10.3390/molecules27196556

**Published:** 2022-10-03

**Authors:** Eugene Sefa Appiah, Perseverance Dzikunu, Nashiru Mahadeen, Daniel Nframah Ampong, Kwadwo Mensah-Darkwa, Anuj Kumar, Ram K. Gupta, Mark Adom-Asamoah

**Affiliations:** 1 Department of Materials Engineering, College of Engineering, Kwame Nkrumah University of Science and Technology, Kumasi AK-448-7139, Ghana; 2 The Brew-Hammond Energy Centre, Kwame Nkrumah University of Science and Technology (KNUST), Kumasi AK-448-7139, Ghana; 3 Nano-Technology Research Laboratory, Department of Chemistry, GLA University, Mathura 281406, India; 4 Department of Chemistry, Kansas Polymer Research Center, Pittsburg State University, Pittsburg KS 66762, USA; 5 Department of Civil Engineering, College of Engineering, Kwame Nkrumah University of Science and Technology, Kumasi AK-448-7139, Ghana,

**Keywords:** biopolymer, supercapacitor, sustainable material, energy density, power density

## Abstract

Supercapacitors may be able to store more energy while maintaining fast charging times; however, they need low-cost and sophisticated electrode materials. Developing innovative and effective carbon-based electrode materials from naturally occurring chemical components is thus critical for supercapacitor development. In this context, biopolymer-derived porous carbon electrode materials for energy storage applications have gained considerable momentum due to their wide accessibility, high porosity, cost-effectiveness, low weight, biodegradability, and environmental friendliness. Moreover, the carbon structures derived from biopolymeric materials possess unique compositional, morphological, and electrochemical properties. This review aims to emphasize (i) the comprehensive concepts of biopolymers and supercapacitors to approach smart carbon-based materials for supercapacitors, (ii) synthesis strategies for biopolymer derived nanostructured carbons, (iii) recent advancements in biopolymer derived nanostructured carbons for supercapacitors, and (iv) challenges and future prospects from the viewpoint of green chemistry-based energy storage. This study is likely to be useful to the scientific community interested in the design of low-cost, efficient, and green electrode materials for supercapacitors as well as various types of electrocatalysis for energy production.

## 1. Introduction

### 1.1. Scientific Stimulus

The generation of energy and its storage involving fossil fuels, as well as global warming, are closely related and contribute to significant environmental problems that threaten human existence. If these problems are not addressed soon, the survival of life on our planet will be in jeopardy for generations to come. Energy storage technologies such as batteries and supercapacitors will play a significant role in the power shift to zero or lower greenhouse gas emissions by connecting renewable energy production to suitable energy storage systems. These energy storage technologies may be used for electric vehicles, and it is expected that over 34 million various types of electric vehicles would be marketed by 2030 [1,2]. This suggests that supercapacitors and batteries have the potential to dramatically reduce greenhouse gas emissions in the electricity and transportation sectors, thereby contributing significantly to the accomplishment of Sustainable Development Goal 7 (clean and affordable energy). Renewable energy sources such as solar energy have been associated with limitations of unavailability in the nighttime and weather conditions. Therefore, energy storage technology must be advanced so that energy is accessible regardless of time or energy source readiness. In this context, batteries and supercapacitors are at the forefront of energy storage devices. Batteries have a high energy density and a constant linear discharge at a high specific voltage and are used as energy backup in commercial applications. They do, however, have disadvantages, such as poor power density, short life cycle, and are explosive.

Nowadays, supercapacitors are emerging to supplement batteries in small but pivotal areas [3,4]. The investigation for the application of supercapacitors as an energy storage device is gearing effectively towards solving energy-related problems as well as climate change. Supercapacitors are superior to conventional energy storage devices (such as a battery) in terms of better power density, good cycle stability, rapid charge–discharge, quick burst power supply, longer lifespan, and functionality in a wide range of operating temperatures, suitable for various electronic and electrical applications [5]. Their poor energy density, however, can be enhanced by synthesizing materials with increased surface-area-to-volume ratios and surface functionalization to boost electrochemical performance. Such a goal is essential for the electrochemical mechanism, as it has a beneficial influence on charge diffusion rate, charge–discharge process, and longer lifespan.

### 1.2. Supercapacitors

Supercapacitors are classified into three primary categories, based on their charge storage mechanism: electric double-layer capacitors (EDLCs), pseudocapacitors (PCs), and hybrid capacitors (HCs) [6,7]. EDLCs are technologically advanced and commercialized devices that use physical adsorption of charges onto the electrode–electrolyte layer. Most commercially available EDLCs use an organic solution as electrolyte and porous carbon as an electrode material. Highly conductive synthetic materials such as carbon nanotubes and graphene are studied extensively; however, these materials are currently not economically feasible due to challenges associated with their synthesis strategies, environmental issues, and high production cost. Moreover, their broader applications are limited due to their lower ionic conductivity and high viscosity.

Charge storage in PCs, in contrast to EDLCs, is primarily accompanied by quick and completely reversible charge transfer (also known as Faradaic reaction). Although it has superior electrochemical performance compared to EDLCs, it is inferior to batteries in terms of energy density. Except for ruthenium oxide, PCs devices have low power densities due to their greater equivalent series resistance; however, their high cost makes them economically unsuitable for practical applications. Materials such as conducting polymers (polyaniline, polythiophene derivatives, and polypyrrole) and transition metal oxides (cobalt oxide, manganese oxide, nickel oxide, and iron oxide) have been used as supercapacitor electrodes [8,9]. Some of them have shown promising possibilities, which require further study.

HCs, on the other hand, can be configured in a variety of ways, including symmetric and asymmetric usage of capacitive and battery-type electrodes. In a symmetric method, composite battery type and capacitive electrodes are typically employed; however, in an asymmetric system, carbon-based capacitive electrodes in combination with pseudocapacitive electrodes are commonly used [5]. HCs exhibit electrochemical behavior that falls between capacitive behavior and battery-like behavior, with a high potential window. The charge storage is enhanced due to the initiation of a reversible redox reaction (battery-type electrode) that compliments the charge storage of the capacitive electrode [10]. Even though these HCs enhance supercapacitors’ energy storage capabilities, they still have poor cycle life, low power densities, and a slow kinetic rate [11].

### 1.3. Bio-Polymers

**Figure 1:**
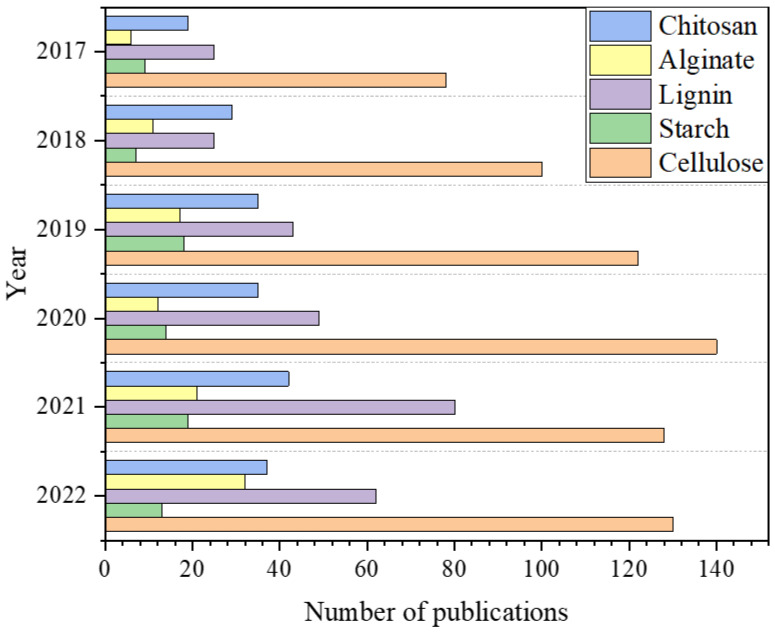
Graphical representation of the published research articles on five (5) different kinds of green biopolymers between 2017–2022 (using Scopus database; date of search 16 September 2022).

Among the several materials accessible for supercapacitor production, current research has focused on natural polymer-based materials for energy storage applications. These materials can assist in increasing sustainability by reducing the usage of nonrenewable and toxic/hazardous resources [12]. As shown in Figure 1, extensive studies on biopolymer-based materials such as cellulose [13], keratin [14], starch [15,16], lignin [17], alginate [18], gelatin [19,20], agar [21], and chitosan [22] have been reported for supercapacitor applications and have indicated a gradual increment over the years.

**Figure 2:**
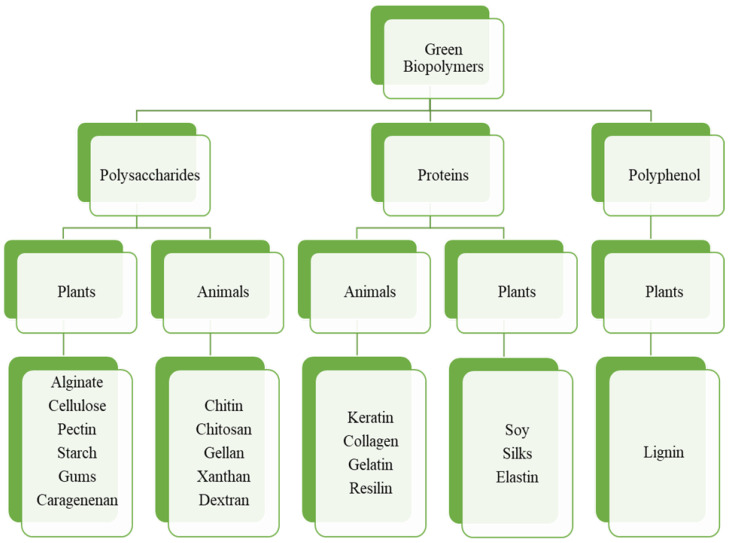
Classification of green biopolymeric materials.

The majority of natural biopolymers are chain-like molecules with repeated units, such as polysaccharides [22] (alginate, cellulose, chitosan, and chitin), proteins [23] (gelatin and silks), and others. Their abundance and excellent properties, such as low cost, biodegradability, renewability, non-toxicity, and textural properties, have encouraged the interest of scientists to investigate their chemical structure, physicochemical properties, and applications [15,24]. Natural biopolymers’ structural properties, in addition to their inherent biological properties, make them great candidates for the synthesis of high-performance composites. As a result, the numerous functional groups (e.g., amine, amino, carboxyl, and hydroxyl groups) from polymer molecular chains endow biopolymers with high hydrophilicity, desirable for the construction of porous materials for energy storage. Furthermore, because of their high reactivity, these functional groups may be modified to fulfill the demands of many specialized functions by altering mechanical characteristics, capacity, crystal structure, electron transfer rates, and solubility as well [1,5,6]. All of the characteristics stated above emphasize the importance of natural biopolymers in the construction of high-performance supercapacitors. Figure 2 illustrates the systematic classification of green biopolymeric materials. This work will appeal to prospective readers including scientists, researchers, and academicians working in the realms of bio-polymers-based materials and manufacturing of effective electrode materials for supercapacitors.

### 1.4. Scope of this Review

This review emphasizes the comprehensive concepts of biopolymers and their synthesis for supercapacitors’ applications. Various synthesis strategies for biopolymer derived nanostructured carbons and their corresponding structure variations have been discussed. Challenges and future prospects from the viewpoint of green chemistry-based energy storage have been highlighted. This study is likely to be useful to the scientific community interested in the design of low-cost and efficient electrode materials for supercapacitors as well as various types of electrocatalysis for energy production.

## 2. Synthesis of Biopolymeric Materials for Supercapacitors

Various synthesis processes such as electrospinning, hydrothermal carbonization (HTC), microwave-assisted activation, sol-gel, and templating have been employed to create biopolymer-based materials for supercapacitor applications. Additional discussions in subsequent sections covers the usage of green biopolymeric materials to prepare hierarchical porous materials for supercapacitors.

### 2.1. Pyrolysis

Pyrolysis is a thermal synthesis technique that happens in the absence, or with a limited fraction, of oxidizing gas, avoiding further oxidation reactions and thereby suppressing carbon dioxide formation. This allows for many reactions, and biopolymeric components are broken down into smaller units. Different products are produced as a result of these reactions. It is critical to highlight that biochar is primarily obtained through biopolymer conversion [11].

The process factors, such as residence temperature and heating rate, influence the final carbon material generated from biopolymers. Temperatures between 600 and 900 °C are the most favorable for producing biochar suitable for supercapacitors. The pyrolysis of biopolymers is not a one-step process. Dehydration, heat degradation, and the formation of a stable carbon structure are usually the first three phases [10]. The pyrolysis process involves synthesizing carbon material from biopolymers to form a large surface area and high porosity. Yang et al. [25] created a porous regenerated cellulose aerogel by dissolving cellulose fibers in a NaOH/urea aqueous solution. The activated RCA-activated carbon aerogel was utilized as an electrode after pyrolysis and activation and displayed a high specific capacitance of 381 F/g in an aqueous electrolyte of 6 M KOH. The carbon aerogel electrode also had a low equivalent series and a low charge transfer resistance, indicating superior capacitive behavior.

Zhuo et al. [26] prepared a porous carbon aerogel as a substrate for the conductive polymer PPy using cellulose as a carbon precursor. The hierarchical porous structure allows for efficient PPy penetration and uniform loading across the carbon network. It ensures quick electrolyte ion transport and PPy accessibility. The synthesized electrode exhibits a high specific capacitance of 388 F/g in 1 M H_2_SO_4_ and excellent cyclic stability, with 93% capacitance retention after 10000 cycles.

The pyrolysis technique increases surface area and porosity, which improves energy storage capacity. Although classic carbonization-activation procedures can produce porous carbons with adjustable SSAs and pore-size distributions, the templates or residues of activation agents must be eliminated after carbonization and activation. As a result, approaches that do not require activation are becoming increasingly appealing. The dehydration and decomposition processes in typical pyrolysis processes of biopolymeric materials result in the emission of gases (e.g., H_2_O, CO_2_, NH_2_, and CO), where the pyrolysis gases function as porogen. The SSA and pore-size distribution of generated carbon material can be finely controlled for supercapacitor use by manipulating pyrolysis parameters such as carbonization temperature, heat ramping rate, and carbonization time.

### 2.2. Electrospinning

**Figure 3:**
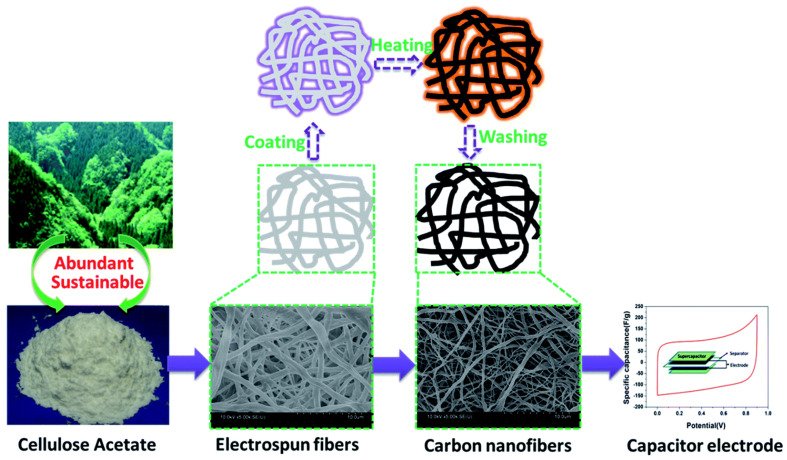
Synthesis route to cellulose acetate-derived carbon nanofibers. Adapted from reference [30]. This article is an open-access article distributed under the terms and conditions of the Creative Commons Attribution (CC BY-NC 3.0) license.

It is a straightforward way to make polymer nanofibers using high voltage [27]. This approach may be used to create fibers from organic polymers while integrating graphene, metal oxides, carbon nanotubes, and other nanomaterials’ [28,29,30] converted cellulose acetate (CA) into porous carbon nanofibers in one step. The electrospun fibers were carbonized and activated in a single process by immersing them in a ZnCl_2_ solution and heating them (Figure 3).

**Figure 4:**
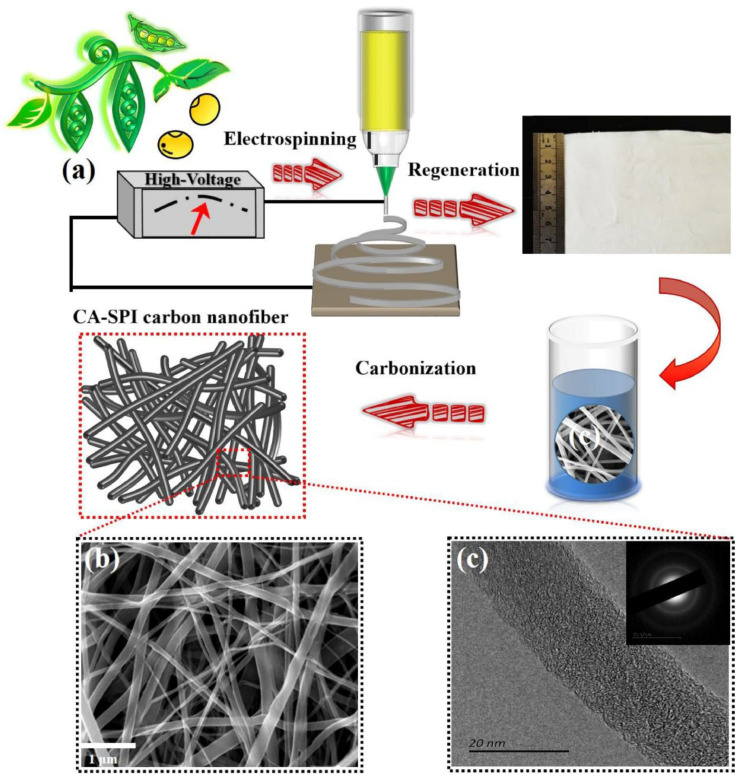
(**a**) Schematic illustration of CA-SPI-CNFs synthesis. (**b**) SEM image of CA-SPI-800 (**c**) TEM image of CA-SPI-800 (Inset is the corresponding SAED pattern). Adapted from reference [29]. This article is an open-access article distributed under the terms and conditions of the Creative Commons Attribution (CC BY-NC-ND 4.0) license.

The activation process compressed micropores, lowering the quantity and expanding the pores. The resulting nanofibers with pore sizes of 0.6 to 1.2 nm, and 1188 m^2^/g active surface area were stable thermally and mechanically. Supercapacitors constructed from the nanofibers showed a high specific capacitance of 202 F/g at 0.1 A/g and constant cyclic performance of 92% after 5000 cycles, demonstrating strong electrochemical stability and reversibility. In another recent study, Wang et al. [29] used electrospinning, regeneration, and carbonization to produce nitrogen-doped carbon nanofibers from cellulose acetate and soy protein isolate, as shown in Figure 4. When assembled into a supercapacitor, a high specific capacitance of 219 F/g was recorded at 0.2 A/g with capacitance retention of 98.9% after 50,000 cycles at 20 A/g.

High-performance supercapacitors can be made using electrospun carbon nanofibers, which has been demonstrated to have considerable potential. Their capacitive performance can be greatly enhanced by increasing the surface area, pore volume, surface wettability, and conductivity of carbon nanofibers. It is clear that the hierarchical porous structure with connected pores will offer substantial active surface areas, rapid ion diffusion, and high-performance electrodes. The rate performance and long-term cycling stability are increased when high conductivity materials, such as graphene and carbon nanotubes, are added into electrospun nanofibers.

Carbon nanofibers can be given a pseudocapacitive property to increase their capacitance, rate capacities, and cycling stability. Examples of this include combining them with heteroatoms, conducting polymers, and transition metal compounds. Despite decades of study on carbon nanofiber electrodes, most carbon nanofiber materials are still in the research and development stage because of their poor production rates and expensive fabrication processes. By using needleless electrospinning processes, precursor nanofiber production will be scaled up, hastening their commercialization. Additionally, using natural resources to create precursor nanofibers could lower the cost of production. The energy storage capacity and capacitor performances will be improved by optimizing the carbonization process, adding a hierarchical porous structure to carbon nanofibers, and boosting electrical conductivity.

### 2.3. Hydrothermal Carbonization

**Figure 5:**
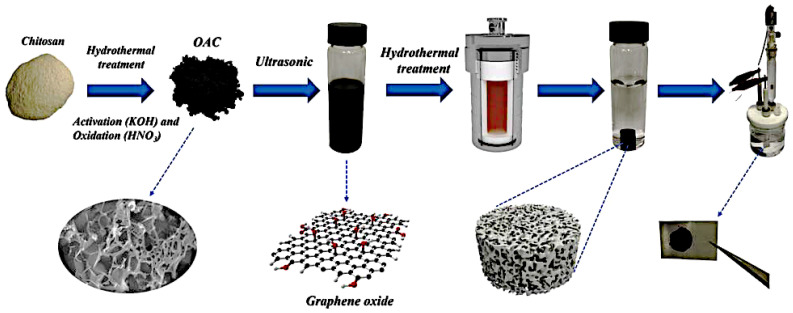
Schematic representation of composite hydrogel synthesis by a hydrothermal reaction. Adapted from reference [32]. This article is an open-access article distributed under the terms and conditions of the Creative Commons Attribution (CC BY-NC-ND 4.0) license.

Hydrothermal carbonization (HTC) is a promising thermo-chemical approach for converting carbon precursors to functional porous materials in a confined environment at low temperature (180–260 °C) and pressure in an aqueous acidic or neutral solution [21]. The aqueous solution, residence time, applied pressure, and temperature all significantly impact the structure–property of the generated biochar. Typically, an activation step is required to enrich the pore structure further and increase the degree of graphitization of biochar. Using this approach, chitosan-derived carbon was generated through hydrothermal carbonization at 200 °C for 2 h and activated with acetic acid. The surface area of the resulting porous carbon was 3532 m^2^/g [21]. Furthermore, Lin et al. [31] discovered that B/N dual-doped porous carbon can be produced using boric acid. The synthesized porous carbon had improved capacitance and electrode stability. The activation procedure began with a 1:2 combination of carbon and boric acid, which was then placed in a Teflon-lined autoclave. For 5 h, the autoclave ran continuously at 160 °C. A capacitance of 316 F/g was recorded. Ren et al. used a one-step hydrothermal approach to produce oxygen-doped activated carbon–graphene composite hydrogels, as shown in Figure 5. In the hybrid hydrogel, high specific surface area-activated carbon derived from chitosan and oxygen-containing groups introduced via a simple room-temperature oxidation strategy with HNO_3_ are assembled into the framework of reduced graphene oxide (rGO) to effectively prevent the restacking of rGO nanosheets and result in high specific surface area and high conductivity of the composite hydrogels, resulting in an excellent energy storage performance. At a current density of 1 A/g, the optimized sample had a high specific capacitance of 376 F/g in 1 M H_2_SO_4_ electrolyte. Furthermore, after 5000 charge–discharge cycles at 10 A/g, the assembled flexible supercapacitor demonstrated optimal cycling stability of 83%.

HTC has been widely reported as one of the most effective methods of producing carbon materials with tunable chemical and physical properties. The HTC carbon structure can be significantly improved (the surface area and pore volume) by combining the HTC with other methods such as templating. Additionally, chemical and physical activation increases specific surface area and creates porosity. Table 1 compares a few samples of HTC-prepared materials to other methods of preparation, as well as their primary porosity properties (surface area) and electrochemical performance.

### 2.4. Microwave-Assisted Activation

Microwave-assisted carbonization technique is an energy-efficient process and can be considered as an alternative to traditional hydrothermal procedures, having the advantages of a short retention period, and homogenous heating. Recently, Jeyabanu et al. [11] used microwave irradiation to create lanthanum nanospheres doped in CuS (La@CuS) at various compositions (La = 0, 1, 3, and 5%). The reduction and oxidation peaks were found at 0.064 V and 0.64 V, respectively, and 100 mV/s scan rate. High specific capacitance of 1329 F/g was obtained for the La = 5% sample at 2 mA current density, while pure CuS with no La doping had a specific capacitance of 1040 F/g at the same current density. The remarkable performance of the La = 5% sample can be due to the addition of pseudocapacitance, which resulted in strong ionic transfer with low internal resistance when compared to the other samples. Li et al. [33] also created nitrogen-doped hierarchical porous carbons with chitosan hydrogels as a precursor and ZnCl_2_ as an activator. The microwave activation of the samples took only 10 min, making it faster and more energy-efficient than conventional approaches. The synthesized N-doped porous material demonstrated a high specific capacitance of 435 F/g at 0.2 A/g in 6 M KOH in a three-electrode setup and outstanding capacitance retention of 71.0% at a high current density of 10 A/g. Furthermore, the designed symmetric supercapacitor exhibits a power density of 50 W/kg, a maximum energy density of 9.4 Wh/kg, and no discernible capacitance loss after 10,000 cycles.

The microwave-assisted activation process can be considered in order to help increase the yield of carbonaceous materials and improve the hierarchical porous structure with less formation and emission of hazardous material. Carbonaceous materials derived from biopolymeric materials via microwave-assisted activation possess excellent properties, such as high SSA, high porosity, and tailored hierarchical superstructure, thus improving the capacitance performance of carbon-based supercapacitors. Thus, the complicated and time-consuming chemical activation followed by the carbonization processes can be simplified into a one-step microwave-assisted activation approach to preparing hierarchically porous carbon.

### 2.5. Template Method

**Figure 6:**
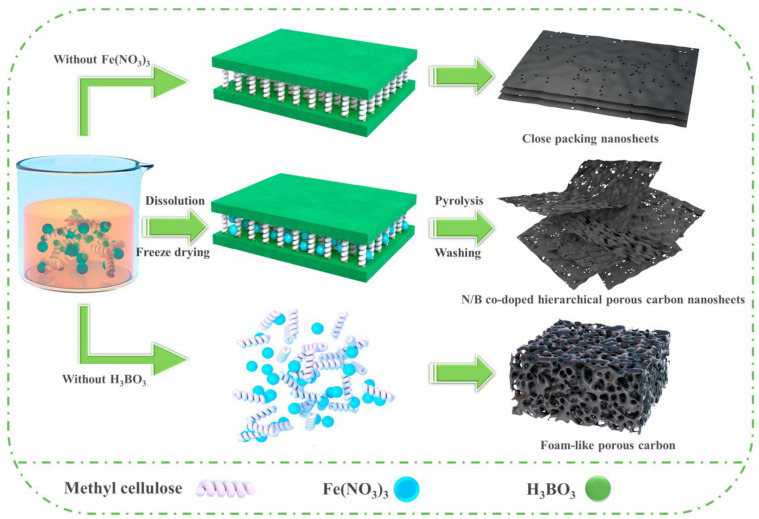
Synthesis of carbon nanosheets via dual template technique. Adapted from reference [36]. This article is an open-access article distributed under the terms and conditions of the Creative Commons Attribution (CC BY-NC-ND 4.0) license.

Template strategies (hard template, soft template, and dual-template) are efficient for preparing porous materials with controlled pore size distribution and moderate morphologies [34,35]. For instance, B and N were doped in carbon nanosheets via a simple dual-template method (Figure 6) using methyl cellulose as a carbon precursor. The resulting carbon electrode displayed a high capacitance of 572 F/g at 0.5 A/g and retained 281 F/g at 50 A/g in an acidic electrolyte, due to the synergistic effects of the large surface area with the hierarchical porous structure, B/N co-doping, and a high degree of graphitization. In addition, after 20,000 cycles in the “water in salt” electrolyte, the symmetric device built with bacterial cellulose-based gel polymer electrolyte delivered a high energy density of 43 Wh/kg and exceptional stability of 97.8% [36].

**Figure 7:**
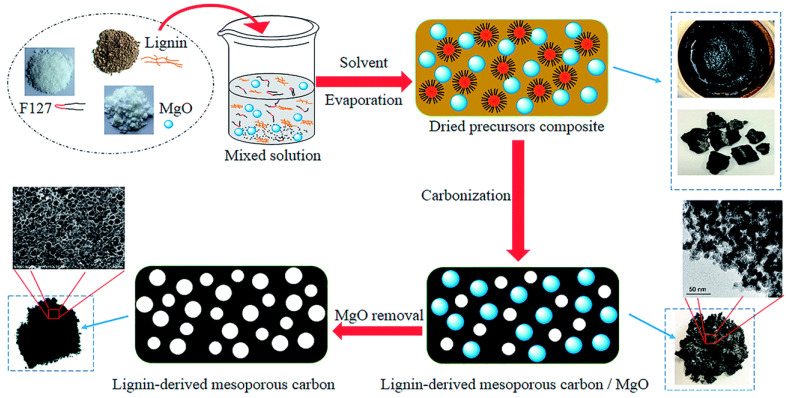
Schematic of lignin-derived mesoporous carbon via a dual-template strategy. Adapted from reference [37]. This article is an open-access article distributed under the terms and conditions of the Creative Commons Attribution (CC BY-NC 3.0) license.

In another work, Song and coworkers [37] also used soft templates (nano-sized MgO and Pluronic F127) to produce hierarchical porous carbon from lignin. Pluronic F127 acts as a dispersant, inhibiting MgO agglomeration, while MgO nanoparticles act as substrates and the principal template through a space-occupying action. Figure 7 depicts the synthesis process, which resulted in an active surface area of 712 m^2^/g and a pore volume of 0.90 cm^3^/g. The resulting carbons’ electrochemical performance as a supercapacitor electrode material was examined. Even though the vacuum environment improved porosity and pore homogeneity, the corresponding carbon did not display superior electrochemical performance to carbon obtained without a vacuum environment.

The template method makes it simple to prepare carbon materials with uniform and tunable pores. The production of the template, however, is an expensive, laborious, and time-consuming process. Another difficulty is being able manage the thickness of the pores, which is essential for regulating the pore diameters of freshly generated carbon materials. In addition, the need for acid or alkali etching to remove inorganic templates limits their usefulness. There are more options to synthesize biopolymeric materials and other carbon sources that cannot be synthesized using other conventional techniques thanks to special templates such as zinc. However, the interplay between the carbon precursor and the templates needs to be thoroughly investigated utilizing various novel templates.

## 3. Recent Advancements in Biopolymers-Derived Materials for Supercapacitors

### 3.1. Keratin-Derived Materials

Keratin is a unique bio-derived polymer with multifunctional features that makes it ideal for use as active material in electrochemical energy storage applications, particularly supercapacitors. Many recent investigations have used keratin in the production of novel green sustainable electrode materials for supercapacitors [43,51,52,53,54]. Despite much new knowledge about the role of keratin in energy storage applications, some researchers [43,55] have also exploited human hair as a keratin source for a similar application. Human hair as a biopolymer has been used in recent times as flexible electrode material for supercapacitors. The rise in wearable energy storage devices has increased the demand for flexible electrode materials to be synthesized for such applications [54]. Human hair possesses properties such as high elasticity, controllable length, and low cost.

**Figure 8:**
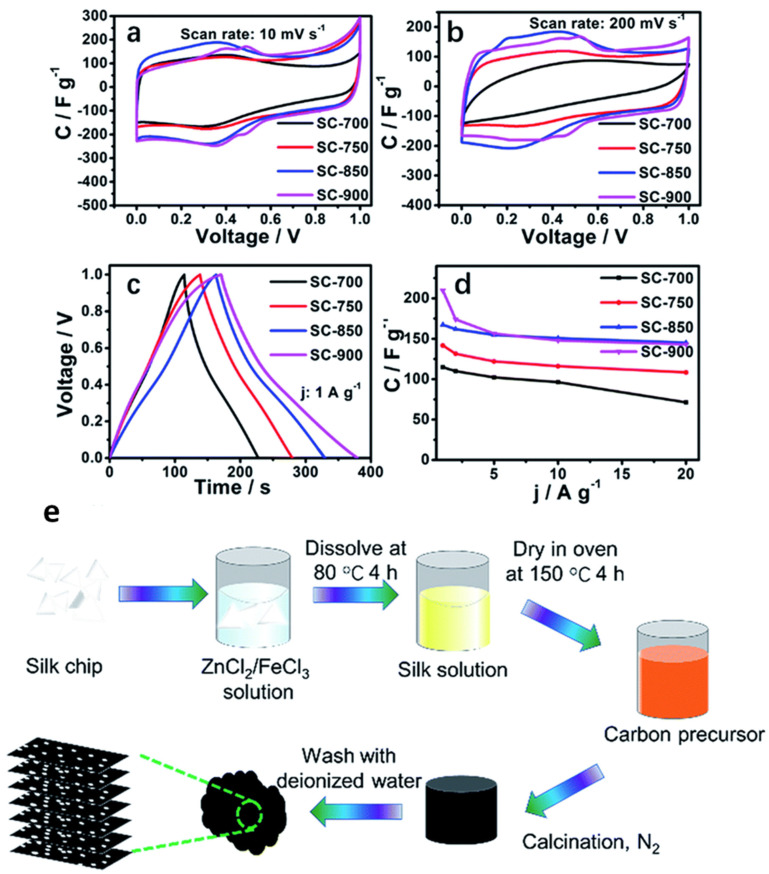
Supercapacitor electrochemical performance evaluation (**a**) CV curves at 10 mV/s. (**b**) CV curves at 200 mV/s. (**c**) GCD curves at 1 A/g. (**d**) Specific capacitances at different current densities. (**e**) Silk synthesis method. Adapted from reference [56]. This article is an open-access article distributed under the terms and conditions of the Creative Commons Attribution (CC BY 3.0) license.

Carbon material can be derived from the keratin in human hair using the microwave-assisted method. An efficient and risk-free microwave-assisted approach was reported by Liu et al. [54] for developing super elastic reduced graphene oxide encapsulating human hair (rGO@Hh) to generate a conductive and high-strength fibrous electrode. The human hair/Ni/rGO/MnO_2_ fiber was created by, first, uniformly coating the human hair with a thin Ni layer via e-beam deposition. Second, after many hours of immersion in GO solution, GO spontaneously assembles on the human hair/Ni fiber. Chemical bath deposition is then used to create honeycomb-structured MnO_2_ on human hair/Ni/rGO fiber. Finally, all-solid-state flexible FSCs are created by carefully twisting the human hair/Ni/rGO/MnO_2_ fiber electrode wrapped in a layer of gel electrolyte and the human hair/Ni/rGO fiber electrode together. The resulting fibers are one-dimensionally coaxial, facilitating electron transport over particle electrode materials. The interwoven rGO@Hh fibers showed an interconnected porous structure with a high specific capacitance of 316 F/g at 1 A/g and strong cycle behavior. Furthermore, rGO@Hh and Ni (OH)_2_/rGO@Hh fibers were used as negative and positive electrodes in a solid-state fiber-based asymmetric supercapacitor (FASC), which showed high energy of 27.6 Wh/kg and a power density of 699 W/kg, respectively. In addition, Zhang et al. [56] reported on simultaneous ZnCl_2_ activation and FeCl_3_ graphitization at varied heating temperatures. This yielded graphitic nitrogen-doped hierarchical porous carbon nanosheets and was employed for supercapacitor application from a readily produced green silk, as shown in Figure 8e. The degree of graphitization and BET surface area increased to their greatest values when the heating temperature was increased from 700 °C to 850 °C, while the nitrogen doping concentration was kept at 2.24 wt%. A porous nanosheet structure offers minimum diffusive resistance and shortens the diffusion pathways. Results from the HR-TEM confirmed the formation of worm-like stripes, indicating the presence of partial graphitization, and many crystal stripes, hinting at high graphitization during the carbonization process.

Carbonized silk with a nanosheet morphology and a large specific surface area (1285.31 m^2^/g) was constructed into a supercapacitor as an electrode material and showed outstanding electrochemical performance with a high specific capacitance of 178 F/g at 0.5 A/g, as shown in Figure 8a-d, and an excellent rate capability, as well as 81% capacitance retention ratio, even at 20 A/g. In an aqueous neutral Na_2_SO_4_ electrolyte, a symmetric supercapacitor with carbonized silk electrodes at 850 °C has exceptional specific energy of 14.33 Wh/kg and a power density of 251 W/kg. Similarly, Sinha et al. [51] described a scalable process for making hierarchically porous, heteroatom-doped activated carbon nanosheets from waste biomass human hair and showed how this carbon might be used as an ultrahigh performance electrode material for supercapacitor applications. The carbon nanosheets (50 to 200 nm size) produced had a hierarchical porous structure with a specific surface area of 1548 m^2^/g. The presence of porous structure is further confirmed with nitrogen adsorption and desorption studies. The well-dispersed graphitic pockets induce the enhancement in conductivity of ions inside the sample and the presence of porosity provides sufficient sites for electrolytic ions to be penetrated. At a current density of 1 A/g, an exceptional specific capacitance value of 999 F/g was obtained. At a power density of 325 W/kg, a maximum energy density of 32 Wh/kg was achieved. Furthermore, at a high current density of 5 A/g, exceptional cyclic stability of 98% capacitance retention was obtained after 10,000 cycles.

### 3.2. Alginate-Derived Materials

**Figure 9:**
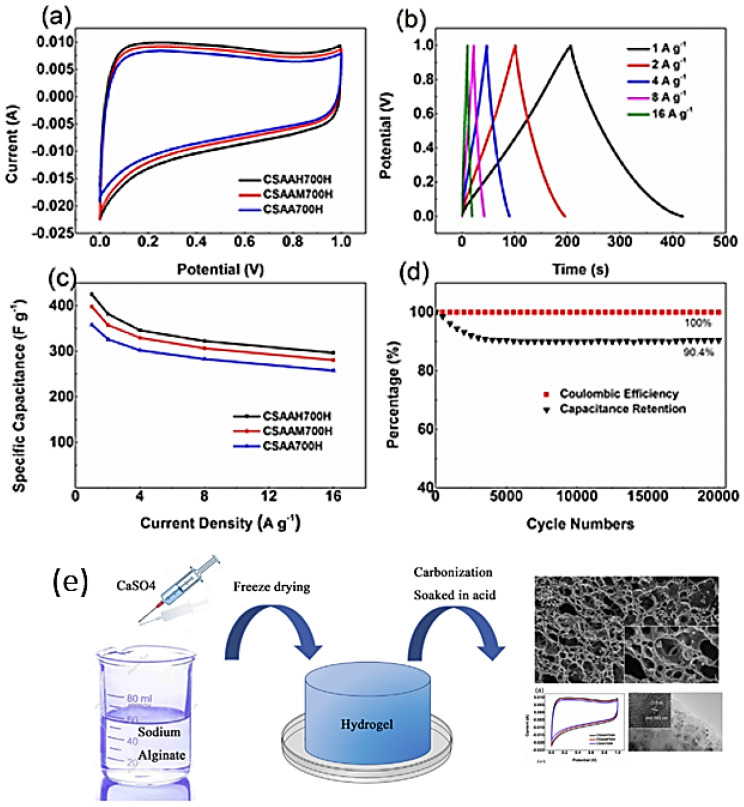
(**a**) CV curves of various synthesized sodium alginate compositions at the scanning rate of 0.01 V/s, (**b**) GCD curves of sodium alginate at different current densities, (**c**) the specific capacitance of various synthesized sodium alginate samples, (**d**) coulombic efficiency and cycle stability at 8 A/g current density, (**e**) synthesis method for sodium alginate. Adapted from reference [67]. This article is an open-access article distributed under the terms and conditions of the Creative Commons Attribution (CC BY-NC-ND 4.0) license.

Alginate is a polyanionic bio-polymer, widely used in various applications for manufacturing energy storage materials from abundant and non-toxic marine biomass resources, such as sodium alginate, potassium alginate, etc. [57]. Recent studies [57,58,59,60,61,62] have indicated that alginate biopolymer-derived biomass carbon materials, in addition to being green, low cost, and renewable, also possess electrochemical properties which make them suitable for supercapacitor applications [63]. Through carbonization of renewable natural alginate, Wang et al. [64] demonstrated a sustainable biomass conversion technique and non-templated strategy for fabricating the macro-, meso-, and microporous carbon aerogel (CA) produced using the sol-gel method. The alginate hydrogel’s gelation process shown in Figure 9e might provide a novel way to make macro- and mesoporous structures. The produced carbon material had a 3D self-supporting core macroporous network with a core diameter of around 2–5 m and thin walls of less than 100 nm, which can operate as electrolyte reservoirs, according to SEM analysis. The presence of many macropores and thin walls was confirmed by the TEM studies. The macropores are approximately 200–300 nm in size (diameter). The high-resolution TEM (HRTEM) image also reveals numerous micropores in the sample as well as an amorphous carbon structure with semi-graphitic domains. The macropores reduce the ion-transport barrier by shortening the electrolyte ion diffusion distance to the active sites. The as-prepared porous CA displays outstanding electrochemical performance, as shown in Figure 9a–d. Moreover, Li et al. [65,66] reported on combining sodium alginate (SA) and dopamine functionalized polypyrrole (DAPPy) nanofibers with borax as a cross-linking agent, yielding a series of multifunctional conductive hydrogels, which was dubbed SA-B-DAPPy. The conductivity of the hydrogel may be increased to 1.33 ± 0.012 S/m by increasing the DAPPy weight ratio to 3.5 wt.%. The adequate stretchability (more than 800%) and immediate self-healing ability of hydrogel frameworks can be attributed to both borate interactions and hydrogen bonds inside the frameworks. The doping process resulted in the formation of a sponge-like structure with rough pore walls. The synergistic effect of the dynamic borate, hydrogen bond cross-linking, and polymer chain entanglements endows the obtained hydrogel with high stretchability (elongation at break greater than 800%), fast self-healing ability (efficient instantaneous electrical and mechanical self-healing within 15 s), and arbitrarily moldable ability.

Similarly, Wei et al. [60] reported on the fabrication of oxygenated N-doped porous carbon as a key step in improving the performance of carbon materials and expanding their applications. Because most methods for the preparation of alginate-derived porous carbon require chemical activation agents and acid washing, synthesizing oxygenated N-doped porous carbon from alginate salts via a simple and low-cost method remains a major challenge. Through carbonization of ammonium alginate aerogel followed by air activation treatment, oxygenated N doped porous carbon with a high surface area was effectively synthesized by Wei et al. [60]. Only the greenest water is used as a solvent in the production of porous carbon, and its volume has a major impact on the porous structure of the carbonization result, which may be further adjusted by air activation treatment. The porous carbon produced from alginate has an interconnected three-dimensional porous network structure. The formation of an interconnected three-dimensional porous network structure is caused by ice sublimation and alginate flexibility. Following air activation, ordered graphene domains with substantially richer porosity architectures can be detected. This finding unequivocally supports the effect of air activation on the porous structure of carbon materials. Due to its rich porous structures and heteroatom doping, the obtained porous carbon exhibits excellent electrochemical performance when used as electrode materials for supercapacitors, such as high specific capacitance (204 F/g at 1 A/g in a two-electrode system), good rate capability, and ultralong cycle life (91.2% capacitance retention after 10,000 charge/discharge cycles). This research might be extended to benefit additional uses of alginate-derived carbon and could pave the way for a novel method of fabricating heteroatom doped porous carbon.

### 3.3. Pectin-Derived Materials

Pectin is a complex group of heteropolysaccharides that make up a significant portion of dicotyledonous plants’ basic cell walls and perform vital functions in their growth and development. Pectin has been extensively researched in a variety of fields, such as electrochemical energy storage [40], food [63], agriculture [64], and medicine [65], due to its excellent properties.

**Figure 10:**
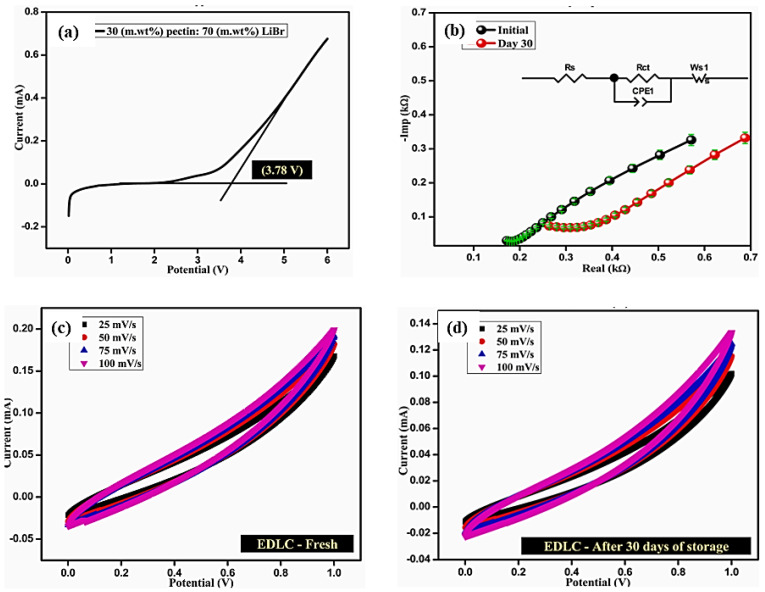
(**a**) LSV plot for Li^+^ complex, (**b**) impedance plots for EDLC before and after storage, fabricated solid-state EDLC CV plot using highest pectin electrolyte (**c**) fresh-Day 1 (**d**) after the storage-Day 30. Adapted from reference [66]. This article is an open-access article distributed under the terms and conditions of the Creative Commons Attribution (CC BY-NC-ND 4.0) license.

Recent studies [41,66,67,68] have shown that pectin biopolymers can be used as an electrode [41,68] and electrolyte [66,67] materials in supercapacitors. Perumal and Selvin [66], for the first time, utilized Li^+^ conducting pectin electrolytes in a solid-state EDLC, manufactured using a solution casting. For additives at a ratio of 30 (m. wt.%) pectin and 70 (m. wt.%) LiBr, the produced pectin electrolytes exhibited a considerable conductivity of 3.44 × 10^−3^ S/cm. This ideal Pectin/LiBr composite material was suggested for the manufacture of solid-state EDLC, as it exhibits an electrochemical capacitance of 19.04 F/g and outstanding reversible performance, as shown in Figure 10. Based on these promising discoveries, research on bio-macromolecular electrolytes offers an advanced foundation for creating resilient energy storage devices, particularly wearable and flexible ones.

The effects of electron-withdrawing–donating groups on the capacitive behavior of p-hydroquinone (PHQ) can be used to improve supercapacitors. The electron-withdrawing group (EWG) and electron-donating group (EDG), in a study by Jie and Ying [69], are sulfonic acids and methoxyl groups, which are the substances of 2,5-dihydroxybenzenesulfonate (DHBS) and 2 methoxy hydroquinone (MHQ), respectively. These substances were derived from pectin and used to improve redox reaction in supercapacitor electrolytes. The use of PHQ, DHBS, and MHQ as redox additives in H_2_SO_4_ electrolyte was demonstrated and two-proton–two-electron redox reactions occur at the electrode–electrolyte interface. When subjected to PHQ, DHBS delivered a larger faradaic impact than MHQ, showing that the EWG is more active than the EDG. Furthermore, all the important redox reactions are kinetically constrained by semi-infinite diffusion. When measured in 1 mol/L H_2_SO_4_, a high energy density of 15.6 Wh/kg was achieved for the DHBS sample with a concentration of 2 mmol/L. Other supercapacitor systems with electron-withdrawing–donating groups would benefit from this current research.

### 3.4. Gelatin-Derived Materials

Gelatin is an alternative biopolymer that is frequently used in the development of supercapacitors’ electrodes, due to its unique biological, gelling, interfacial, and viscoelastic characteristics, and is also used in the energy storage and conversion sectors [1,24,70]. Based on these characteristics, Sun et al. [9] prepared a nanocomposite electrode synthesized by embedding tungsten disulfide (WS_2_) nanoparticles in gelatin-derived hierarchical porous carbon. Honeycomb-like porous nanostructures were formed after washing out the salt. A number of WS_2_ nanoparticles were dispersed and anchored on the carbon sheet. The shapes of these particles were irregular, and the sizes ranged from ~20 nm to ~100 nm. Due to its structural advantages, the generated nanocomposite electrode displayed significantly improved electrochemical characteristics. It had a high capacitance value of 1305.5 F/g as an electrode material at a current density of 0.2 A/g. Even after 3000 cycles at 10 A/g, the nanocomposite maintained a high capacitance of 482.6 F/g and preserved 94% of its original capacitance. Activated carbon was used as the anode and WS_2_ nanocomposite as the cathode in the construction of an asymmetric capacitor. At 0.1 A/g, a capacitance of 135.9 F/g, an energy density of 27.2 Wh/kg, and a power density of 59.9 W/kg was attained. Furthermore, gelatin/starch/TiO_2_ nanocomposite, which was synthesized by hydrolysis and microwave activation, showed good supercapacitive performance at a negative voltage window. A well dispersed TiO_2_ NPs dispersed within the crystalline starch nanoparticles and oval shaped nano-gelatin NPs was formed. The nano-starch biopolymer serves as a compatible agent due to the similar bond structure it has with gelatin, and it will also help to optimize its low retention capacity. The TiO_2_ does not support dendrite growths, so it alleviates volumetric variations, thereby reducing any form of nucleation overpotential during charging. At 5 mV/s, the three-electrode configuration achieved a high specific capacitance of 808 F/g, as well as an energy density of 208.3 Wh/Kg, and capacitance retention of 95% after 5000 cycles. The fabricated supercapacitor has a capacitance of 617 F/g and capacitance retention of 92% after 5000 cycles [71]. The utilization of nano level gelatin and starch for the fabrication of supercapacitor electrodes with superior electrochemical performance at negative volt-ages for possible applications in signal control systems and smart switches, as well as devices for future 4IR applications, is what makes this work unique. It serves as a foundation for the development of symmetrical supercapacitors that can readily switch polarities in places that require both positive and negative voltages. This innovative nano-form of gelatin and starch biopolymers outperforms their bulk counterparts in terms of adjustable transport characteristics and distinctive surface chemistry.

### 3.5. Lignin-Derived Materials

Lignin is a plentiful and easily accessible biomass regarded as a one-of-a-kind sustainable organic carbon source [72]. Lignin is a macromolecule with an exceedingly complicated three-dimensional reticulated molecular structure [6,73], containing 40–60% carbon, and is an excellent precursor for porous carbon [70]. Many features distinguish lignin-based porous carbon materials, including large active surface area, superior electrical conductivity, an interconnected pore structure, and high porosity. Porous carbon compounds based on lignin have several uses in supercapacitor applications [74,75]. Schlee et al. [76] spun the lignin fiber using kraft lignin. Thermal stabilization, carbonization, and CO_2_ activation were used to create lignin fiber with a self-supporting microporous structure. Because of the extraction, it was feasible to electrospun fibers from pure lignin without the use of any additional synthetic high molecular weight polymer, such as PVA. The resulting fiber mats are made up of randomly intertwined cylindrical fibers. The average diameter of the as-spun fibers is 769 nm, which is rather large when compared to prior studies demonstrating that intermolecular interactions must occur [33,66]. Furthermore, the high polymer concentration in the solution (50 wt%), combined with the lack of additives (salts or co-polymers), results in fibers with large diameters [75]. The non-activated fiber is dense, whereas the CO2-activated fiber has more porosity and hence seems less dense. As a result, when evaluated in 6 M KOH electrolyte, the CO2-activated fiber mats demonstrated a high specific gravimetric capacitance of 155 F/g at 0.1 A/g, great rate capability of 113 F/g at 250 A/g, and good capacitance retention of 94% after 6000 cycles. As a result, it is possible to conclude that lignin is a suitable precursor for the production of microporous, oxygen functionalized carbon fibers for use as free-standing electrodes in aqueous supercapacitors.

**Figure 11:**
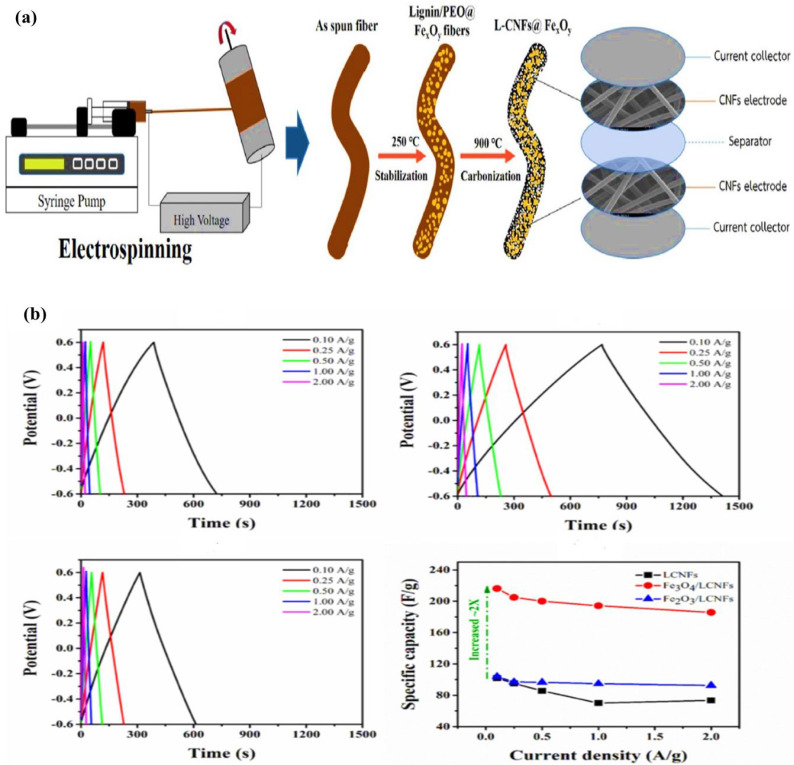
(**a**) Schematic illustration of the fabrication for lignin-CNF-Fe_3_O_4_ composite. (**b**) Galvanostatic charge–discharge curves of L-CNFs, L-CNFs@Fe_3_O_4_, L-CNFs@Fe_2_O_3_, and plots of specific capacitance versus the current density of composite materials. Adapted from reference [28]. This article is an open-access article distributed under the terms and conditions of the Creative Commons Attribution (CC BY-NC-ND 4.0) license.

Although some researchers have indicated that employing lignin as a single spinning solution through electrospinning and carbonization can effectively create nanofibers, in most situations, pure lignin electrospinning produces spray rather than fibers. As a result, additional high molecular weight polymers with strong spinnability properties, such as PAN, PEO, PVA, and PVP, must be added to the spinning solution during the electrospinning process [75]. In that regard, Butnoi et. al. [28] used a one-step electrospinning process to create a flexible nanofiber composite comprising lignin and nanofibers containing iron oxide nanoparticles, as shown in Figure 11a. Because of visible porosity and lumps, the surface of the doped nanofibers was rougher. This might be due to iron agglomeration and phase separations between the polymer and the iron precursor. The as-synthesized materials have well-retained carbon nanofibers in three-dimensional networks of nonwoven mats with randomly oriented nanofibers. The 10 wt% iron doped fibers had a higher average fiber diameter of 625 ± 133 nm. At a current density of 0.1 A/g, the nanofiber-Fe_3_O_4_ electrode had a specific capacitance of 216 F/g and retained 97% of its capacitance after 1000 cycles at 1 A/g (Figure 11b). It should be emphasized that nanofibers generated by electrospinning from lignin have a high aspect ratio, a high specific surface area, high electrical conductivity, and good mechanical properties, making them a potential candidate material in the field of energy storage. From the research conducted by [77] and [28], the use of metal salt additions improves the pore structure and overall performance of the material.

A completely different approach based on spray drying and carbonization was employed by Cao [47] to prepare hollow carbon spheres. Kraft lignin was used as a carbon precursor, while potassium hydroxide (KOH) was used as an activator. The porous carbon particles produced have a surface area of 2425 m^2^/g and a mixture of hollow and spherical morphologies. The diffraction peaks from the (0 0 2) planes of graphitic carbon and (1 0 0) planes of the graphite lattice confirmed the formation of amorphous porous structures that are partially graphitized due to the simple carbonization process. When employed as a supercapacitor electrode, 31.8 F/g at 0.2 A/g was measured, which is greater than the 29.2 F/g produced from commercial activated carbon Kuraray YP-50F.

### 3.6. Cellulose-Derived Materials

Cellulose is a popular biopolymer utilized in supercapacitor applications, derived from either plants or microorganisms [13]. Bacterial cellulose precursors have various benefits over classical precursors, including a more porous structure, a bigger surface area, superior biodegradability, large-scale cellulose production, and a high concentration of hydroxyl groups [6]. Due to its robust interaction with various added chemicals and the finely thinner morphology of bacterial cellulose compared to plant cellulose, these advantages transform bacterial cellulose into a tunable flexible scaffold appealing biomaterial that attracts widespread interest in forming a highly versatile three-dimensional carbon nanomaterial [78,79].

Hao et al. [80] employed bacterial cellulose to produce a hierarchical 3D porous carbon nanofiber network. When used as a binder-free electrode, the carbon nanofibers had a high surface area of 624 m^2^/g and good electrochemical performance. The TEM results also indicated a linked 3D porous network architecture made up of 20–50 nm nanofiber networks and developed porosity. Notably, such short nanofibers might give a high surface-to-volume ratio to boost SSA for charge accommodation during the charge–discharge process, while the unique interconnected porous network architecture will promote quick electron transfer/ion diffusion along 3D directions. According to high-resolution TEM (HRTEM), the nano-fibers are mostly formed of aligned graphitic layers (002) with a lattice distance of 0.34 nm and turbostratic carbon. The graphitic structure promotes ion–electron movement. In a three-electrode system with 0.5 A/g current density in 6 M KOH electrolyte, the as-prepared electrode had a maximum specific capacitance of 302 F/g. The symmetric supercapacitor created has a specific capacitance of 184 F/g at 0.25 A/g in aqueous electrolyte and remarkable capacitance retention of 98% after 5000 cycles. Mo et al. [81] also used sheet cellulose to create graphene-like porous carbon with a hierarchical pore structure. At 1 A/g in 6 M KOH aqueous electrolyte, a high specific surface area of 2045 m^2^/g was produced, resulting in gravimetric capacitance of 353 F/g and volumetric capacitance of 309.7 F/cm^3^. In an ionic liquid electrolyte in acetonitrile, the symmetric supercapacitor had gravimetric and volumetric specific energy densities of 120.1 Wh/kg and 80.4 Wh/L, respectively. In addition, Luo et al. [82] also created N-doped hierarchically porous nanocarbons from microcrystalline cellulose and tandem thermal treatment in CO_2_, N_2_, and NH_3_. It was possible to produce a carbon sponge with hierarchical pores and bi-modal (micro- and meso-porous) architectures. The stacked slice layers on the carbon surface made the carbon blocks appear fluffier, aggregating into 3D porous channels and voids. The porous cross-linked network is seen in the amorphous and extremely disordered carbon structures of the carbon material, which is filled with homogeneously scattered micro-mesopores. The pore volume of the optimized pyrolytic carbon was 1.6291 cm^3^/g, and the specific surface area was up to 3278 m^2^/g. According to electrochemical testing, the synthesized carbon delivered a specific capacitance of 234 F/g at 0.5 A/g and stayed at 201 F/g at 5 A/g, suggesting a strong storage capacity for supercapacitor applications.

### 3.7. Starch-Derived Materials

**Figure 12:**
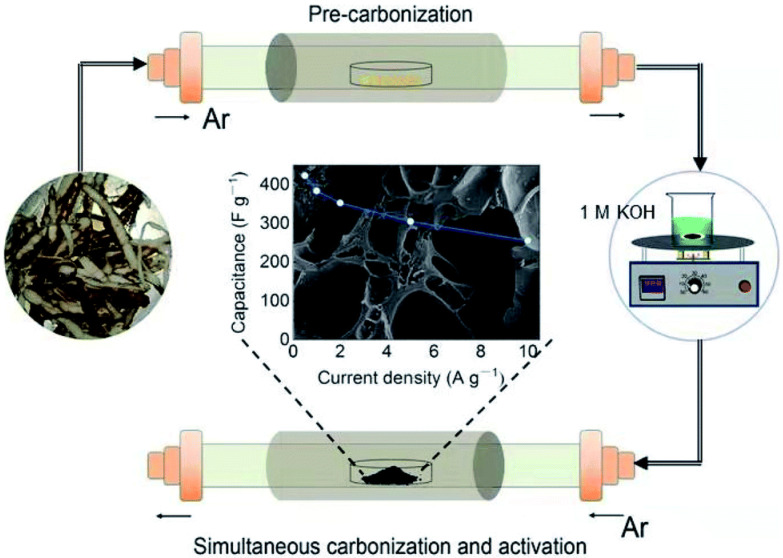
Schematic illustration of YPC derived from yam waste. Adapted from reference [85]. This article is an open-access article distributed under the terms and conditions of the Creative Commons Attribution (CC BY 3.0) license.

Polysaccharides and other similar biopolymers such as starch can be used as green electrodes in supercapacitor applications rather than just as binders and electrolytes, according to many recent studies [83]. A starch-derived porous carbon, as green electrode material for a supercapacitor, was reported by Pang et al. [84]. The XRD patterns indicated an increasing regularity of crystal structure resulting in better layer alignment. The morphology also indicated an irregular blocky shape structure with rough outer surface, and the particles were around 1–5 μm in size. The material displayed a specific capacitance of 144 F/g at a current density of 0.625 A/g in 6 mol/L KOH electrolyte and an energy density of 19.9 Wh/kg at a power density of 311 W/kg. Furthermore, Li et al. [85] used two-step carbonization and KOH chemical activation technique to create yam waste-derived 3D hierarchical porous carbon, as shown in Figure 12. The optimized sample has an ultra-high specific surface area of 2382 m^2^/g, a pore volume of 1.11 cm^3^/g, and simultaneous O-N co-doping. The resulting porous carbon has a pseudo-honeycomb-like 3D net structure with a pore diameter of about 50 μm and a thickness of 1–10 μm. When the prepared sample is employed as an electrode material for a supercapacitor, its structure promotes effective ion diffusion. Because of these unique characteristics, the optimized material has a high gravimetric capacitance of 423.23 F/g at 0.5 A/g, an impressive rate capability at 10 A/g, and outstanding cycling durability, with 96.4% capacity retention at a high current density of 10 A/g after cycles in 6 M KOH in a three-electrode system.

**Figure 13:**
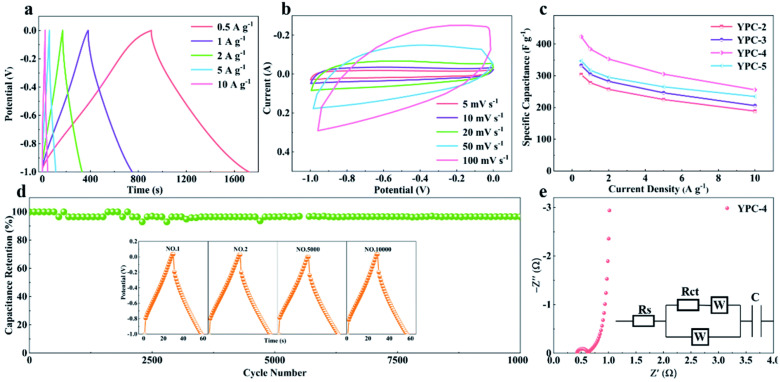
Electrochemical performance of YPC-4. (**a**) GCD profiles at different current densities; (**b**) CV curves at different scan rates; and (**c**) specific capacitance values of YPC-4 at different current densities. (**d**) Cycling performance of the YPC-4 electrode over 10,000 cycles at 10 A g^−1^; the inset shows the GCD profiles at different cycles. (**e**) Nyquist plot of the YPC-4 electrode; the inset shows the corresponding equivalent circuit model. Adapted from reference [85]. This article is an open-access article distributed under the terms and conditions of the Creative Commons Attribution (CC BY 3.0) license.

Furthermore, the built symmetrical supercapacitor delivers a huge capacitance of 387.3 F/g at 0.5 A/g in 6 M KOH electrolyte. In 1 M Na_2_SO_4_ electrolyte, it likewise exhibits high specific energy of 34.6 Wh/kg when the specific power is 200.1 W/kg, and commendable specific energy of 8.3 Wh/kg when the specific power is 4000 W/kg, as shown in Figure 13. As a result, this research can be used to develop high-performance electrode materials for supercapacitors.

Unlike Ruibin et al. and Pang et al., the following researchers focused on the use of different synthesis methods to produce their starch-based carbon materials and recorded different electrochemical properties. Chen et al. [86] utilized a two-step procedure for the fabrication of ultrathin carbon nanosheet-supported Ni quantum dot hybrids (C-Ni-QDs). TEM was used to examine the morphologies of C-Ni-QDs hybrids. The sample has a large number of Ni QDs that are equally distributed on the surface of a carbon nanosheet. The carbon nanosheets are transparent when exposed to electron beams, indicating their ultrathin nature. Furthermore, HR-TEM demonstrates that Ni QDs are approximately 5–10 nm in size with low agglomeration, which can give additional surface-active sites. The hybrid materials produced had a high specific capacitance of 1120 F/g at 2 A/g and outstanding rate capability with 81.3% retention ratio at 20 A/g. In addition, the C-Ni-QDs hybrids showed excellent cycling stability, with 97% capacitance retention after 2000 cycles. Additionally, Saliu et al. [87] reported on an as-prepared A-TiO_2_ and U-TiO_2_ which were achieved through the synthesis of TiO_2_ on an activated and un-activated starch template, respectively, whiles the synthesis of TiO_2_ without a template is unsupported-TiO_2_. The utmost result was A-TiO_2_, which has 72% anatase and 28% rutile and has a specific capacitance of 388 F/g, an energy density of 194 Wh/kg, a power density of 4473 W/kg, and 99% retention capacity after 20,000 cycles.

### 3.8. Chitosan-Derived Materials

Chitosan is the subject of extensive study and development because it is seen as a material with enormous potential for structural alterations to impart desired qualities and functionalities in the future. Recently, Wei et al. [88] reported having successfully formed a sandwich-like structure (CPCM/MXene) by incorporating Ti_3_C_2_T_x_ MXene into chitosan-based porous carbon microsphere (CPCM) through electrostatic contact. Because porous carbon microspheres generated from chitosan have a 3D honeycomb structure, incorporating Ti3C2Tx into carbon microspheres has no effect on this structure. Furthermore, Ti3C2Tx nanosheets bind to the surface of the porous carbon microspheres, thereby preventing stacking and forming a sandwich-like structure. Lv et al. [89] and Xiao et al. [90] effectively synthesized nitrogen and sulfur co-doped porous chitosan hydrogel-derived carbons (CHC-SK) and N, P-co-doped porous carbon materials (NPPCs), using one-step carbonization technique and new phytic acid-induced self-assembled chitosan aerogel synthesis, followed by pyrolysis/activation respectively.

**Figure 14:**
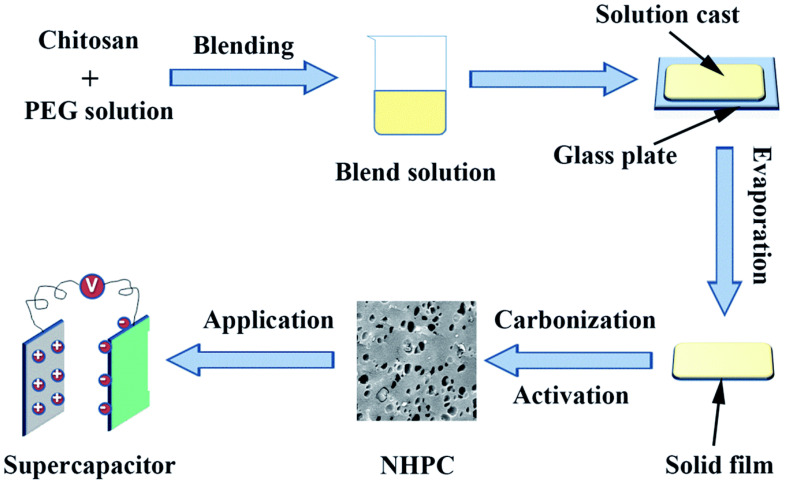
Schematic illustration of the preparation of NHPC materials. Adapted from reference [91]. This article is an open access article distributed under the terms and conditions of the Creative Commons Attribution (CC BY 3.0) license.

In another work, Ba et al. [91] concentrated on nitrogen-doped hierarchical porous carbon (NHPC) materials that were manufactured employing a chitosan–polyethylene glycol (PEG) blend as the raw material and a simple carbonization-activation procedure, as shown in Figure 14. Chitosan was used as a nitrogen-containing carbon precursor, and low-cost, large-scale commercial PEG was used as a porogen in this approach. The ratio of chitosan and PEG had an impact on the physical and electrochemical properties of the NHPC that resulted. The sample obtained by the 3:2 ratio had a large specific surface area (2269 m^2^/g), mild nitrogen doping (3.22%), and a well-structured pore structure. NHPC exhibits an amorphous structure, and some locally ordered structure could be observed, indicating partial graphitization of the synthesized materials. At a current density of 1 A/g, it showed a high specific capacitance of 356 F/g in 1 M H_2_SO_4_ and 271 F/g in 2 M KOH, and over 230 F/g was retained at a current density of 20 A/g in both electrolytes.

**Figure 15:**
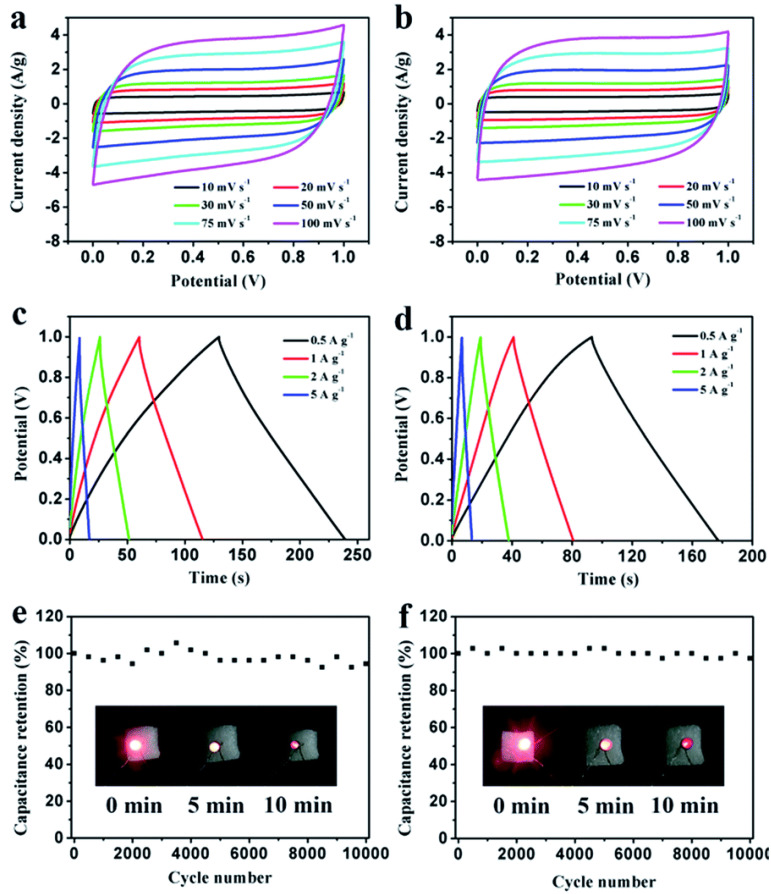
Electrochemical performance of PEG-40% symmetrical supercapacitors in 1 M H_2_SO_4_ and 2 M KOH, respectively. (**a**,**b**) CV curves at different scanning rates; (**c**,**d**) GCD curves at different current densities; (**e**,**f**) cycling stability at a 1 A/g. Adapted from reference [91]. This article is an open access article distributed under the terms and conditions of the Creative Commons Attribution (CC BY 3.0) license.

In addition, as shown in Figure 15, the built symmetric supercapacitors have outstanding cycling stability, with 94% (in 1 M H_2_SO_4_) and 97% (in 2 M KOH) retention after 10,000 cycles at 1 A/g. These findings suggest that a chitosan–PEG blend can be used as a unique and suitable precursor for the preparation of low-cost NHPC materials for high-performance supercapacitors. Nayak et al. [92], unlike the other researchers, obtained Ag-Zirconia nanocomposite material using a green synthesis approach. The Ag-Zirconia nanocomposites outperformed the pure Zirconia in terms of specific capacitance, which is 256 F/g as against 193 F/g at a current density of 1 A/g. Furthermore, a supercapacitor device was constructed employing the synthesized nanocomposites and activated carbon, which had an enhanced energy density of 31.94 Wh/kg at a power density of 500.86 W/kg. The device’s stability was also noted to be outstanding, with 89% retention capacity after 2500 cycles at 10 A/g.

### 3.9. Chitin-Derived Materials

Chitin is a naturally occurring polymer found in abundance around cellulosic biomass [84,85,86]. Deproteinization [87,88,89,90,91] and demineralization [92,93,94,95,96] are used to crude chitin to make it usable [97]. Recent research articles focused on the fabrication and electrochemical performance of the use of chitin-based materials for energy storage.

**Figure 16:**
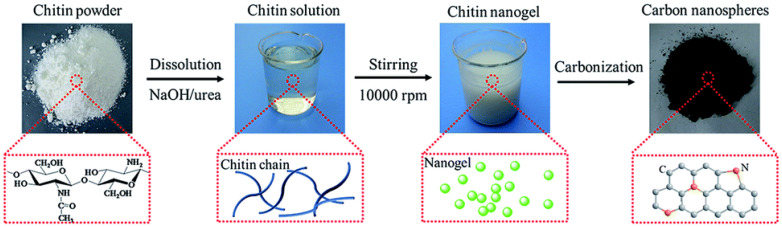
Representation of the formation process for chitin-derived nanospheres. Adapted from reference [96]. This article is an open access article distributed under the terms and conditions of the Creative Commons Attribution (CC BY 3.0) license.

Wang et al. [93] and Shang et al. [94] both described a new method for producing heteroatom-doped hierarchical porous carbon (HPC-700) and biobased N-doped hierarchically porous carbon (N-HPC) electrode from chitin/KMnO_4_ activating agent and marine crustacean derivatives/chitin nanofibers (ChNF), respectively. As a consequence, the HPC-700 electrode had an ultrahigh specific capacitance of 412.5 F/g at 0.5 A/g, outstanding electrochemical stability (only 0.4% loss after 10,000 cycles), and a high energy density of 9.67 Wh/kg, whereas the findings of N-HPC electrodes revealed that N-HPC electrodes had a capacitive performance of 128.5 F/g at 0.2 A/g and a very good electrochemical stability even after 5000 cycles. Zheng et al. [95] made N-doped porous carbon nanospheres by direct pyrolysis of chitin nanogels, which were easily created by mechanical agitation caused by sol-gel transition of chitin solution in NaOH/urea solvent, as shown in Figure 16. The carbon nanospheres are assembled into an interconnected framework with partial graphitized structure and an adjacent interlayer distance of ~0.3 nm.

**Figure 17:**
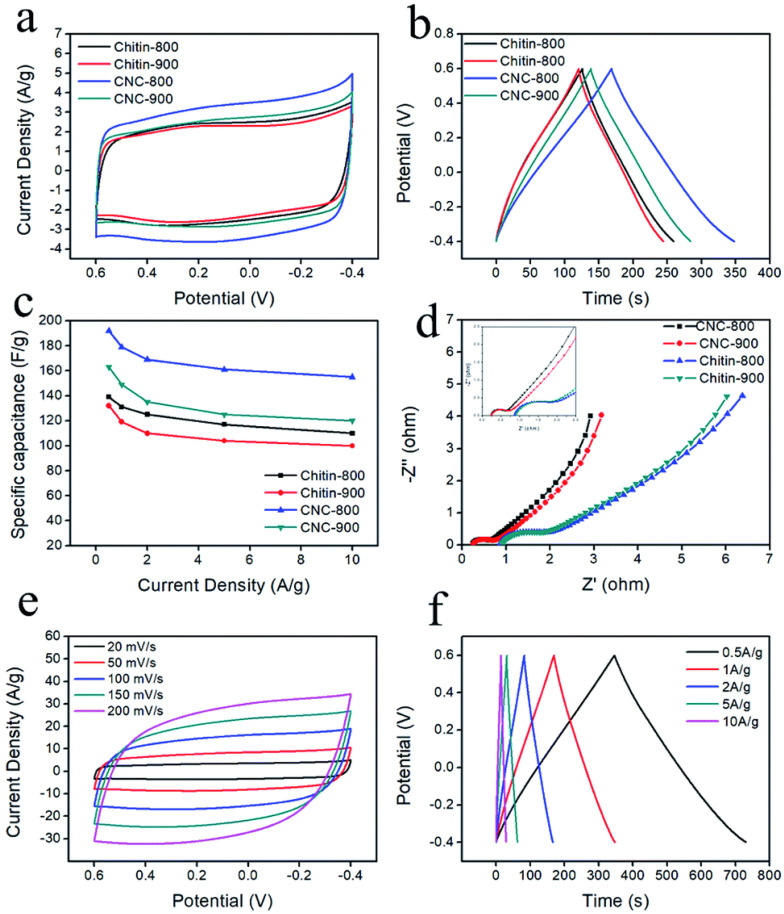
(**a**) CV curves of carbons at a scan rate of 20 mV/s; galvanostatic charge–discharge curves (**b**) at a current density of 1 A/g; specific capacitance versus current density (**c**); EIS analysis (**d**); CV curves (**e**) of CNC-800 at different scan rates in 1 M H_2_SO_4_ aqueous solution; galvanostatic charge–discharge curves (**f**) at different current densities. Adapted from reference [96]. This article is an open access article distributed under the terms and conditions of the Creative Commons Attribution (CC BY 3.0) license.

The carbon nanospheres that emerged had structured micropores (centered at 0.6 nm) and a high BET surface area of up to 1363 m^2^/g, which was significantly higher than that of raw chitin carbons (600 m^2^/g). Furthermore, the carbon nanospheres had a nitrogen concentration of 3.2% and good conductivity. As a result, supercapacitor electrodes made from carbon nanospheres pyrolyzed at 800 °C demonstrated a specific capacitance of 192 F/g at 0.5 A/g current density and outstanding rate capability. As shown in Figure 17, N-doped porous carbon nanospheres showed outstanding cycling stability in both aqueous and organic electrolytes, as well as an outstanding energy density of 5.1 Wh/kg at a power density of 2364.9 Wh/kg when assembled in a symmetrical two-electrode cell. This research presents a new and effective process for making N-doped porous carbon nanospheres directly from chitin, demonstrating the enormous potential of using abundant polymers found in nature for energy storage.

### 3.10. Collagen-Derived Materials

Collagens are the most common family, with more than 20 distinct collagen types recognized currently [97,98,99,100]. Collagens are essential for the production of extracellular matrix fibrillar and microfibrillar networks, basement membranes, and other extracellular matrix structures [101,102]. The extracellular matrix nature of collagen gives it a larger surface area for super capacitive applications [103,104]. Lei et al. [105] reported on a feasible and simple approach for synthesizing Mn-doped N-containing carbon compounds using collagen waste for supercapacitor applications. The metal ions were chelated with bayberry-tannin-immobilized collagen fiber, followed by a carbonization procedure, to produce Mn-doped N-containing carbon. The highest specific capacitance value for the Mn/N-C-x material (where x is the carbonization temperature) was 272.62 F/g in 1 M KOH electrolyte solution with a highly obtained nitrogen content at a carbonization temperature of 800 °C. However, at a carbonization temperature of 1000 °C, the Mn/N-C-x material recorded the lowest specific capacitance value of 240.27 F/g resulting from the decrease in nitrogen content. The rate capability of the Mn/N-C-800 was 72.21% from 1 to 20 A/g, and it remained at 81.4% after 6000 cycles. In another work, Yu et al. [106] stated the use of sol-gel, freeze-drying, carbonization, and KOH activation multistep processes to make nitrogen-doped carbon aerogels by employing sodium carboxymethyl cellulose (ferric trichloride and collagen), as a cross-linking agent, and nitrogen sources, respectively. Carbon aerogels with well-developed porous three-dimensional morphologies, large specific surface areas, and outstanding magnetic characteristics were discovered. The samples all have the characteristic crosslinked 3D network structure. Clearly, the amount of collagen introduced enhanced the lamellar thickness. This could be because the high collagen content slowed the cross-linking rate and lengthened the gel development stage, resulting in the creation of comparatively thick layers after the freeze-drying and carbonization processes. In a 6 M KOH electrolyte, the CA-N_0.5_ demonstrated a specific capacitance of 185.3 F/g when used as an electrode material with a current density of 0.5 A/g. After 5000 charge–discharge cycles, the specific capacitance retention was 90.2%, showing outstanding cycling stability. To access the electrochemical performance of the various biopolymeric materials stated earlier, Table 2 lists the electrochemical performances of several biopolymeric materials.

## 4. Challenges of Biopolymer-Based Materials for Supercapacitor Applications

One aspect of technology that is becoming increasingly essential is choice of materials for fabrication of energy storage devices. Over the years, there has been major variations in the materials used for electrolyte and electrode. Recently, significant achievements in the key aspects of energy storage devices that includes electrode–electrolytes materials were unveiled. The achievements lie in the utilization of environmentally friendly, inexpensive, flexible, and lightweight electrode–electrolyte materials for energy storage. Several natural precursor materials have been explored for flexible wearable devices. Biopolymers are one of the natural materials used for electrode–electrolyte of the flexible wearable devices. Biopolymers are currently at the verge of becoming the most suited precursor material for electrode–electrolyte materials due to its excellent properties such as high flexibility, lightweight, and environmental friendliness.

Carbon-based materials are a family of the most promising and attractive electrode materials, and they offer many advantages that make them perfect for supercapacitor applications. They are earth-abundant, non-toxic, and inexpensive materials, yet have good operational performance. Despite the impressive properties exhibited by biopolymer derived electrode materials, there is still room for improvement on the microstructures in order to achieve higher energy and power densities. For instance, the size, structure, morphology, and surface properties of generated carbons may vary greatly depending on the developing environment and subsequent composition of natural biopolymer sources received from distinct regions. As a result, it is challenging to replicate the electrochemical characteristics of carbons. A price variance in the market may result from the widespread usage of commercially valuable biopolymers (such as keratin, chitosan, chitin, and starch) to manufacture carbon electrode materials, outweighing any potential economic benefits. Additionally, necessary pre-processing tasks, such as reducing biopolymers to fine powders and eliminating impurities, are time-consuming and difficult. Some fabrication techniques also call for harsh environmental conditions such as high temperatures and corrosive chemicals. Therefore, it is crucial for researchers to concentrate on low-cost techniques for isolating biopolymers, such as keratin, starch, gelatin, alginate, chitosan, and so forth, from their natural sources, enabling hassle-free advancement in the field of biopolymer-based electrode materials.

More importantly, biopolymer-based carbons typically have disordered pore morphologies and a wide range of pore sizes. The rate performance and power density are constrained by the difficulty of precisely tuning pore parameters such as shape, structure, and regularity. Furthermore, understanding the different interactions that the biopolymer contributes to (covalent bonding, hydrogen bonding, van der Waals interaction, etc.) is crucial since they improve the mechanical and electrical characteristics of the resulting biopolymer-based electroactive material.

In this regard, it is imperative to design high-performance biopolymer-based materials as an electrode and electrolyte for solid-state and flexible supercapacitor applications.

## 5. Conclusions and Future Outlook

**Figure 18:**
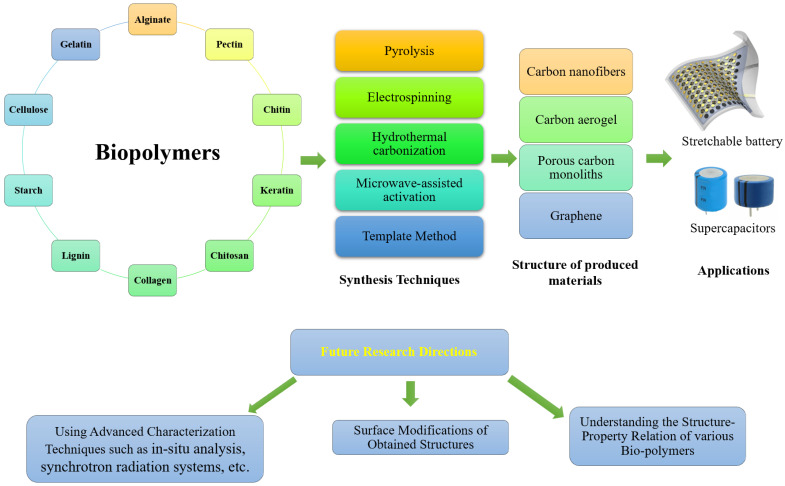
Summary of the various bio-polymers, synthesis, produced structures, applications, and future research directions.

The goal of this review was to provide an overview of the most current developments in the use of biopolymer-based materials for supercapacitor applications. Using sustainable and low-cost green biopolymers as raw precursors to make porous electrodes may assist to lessen dependency on nonrenewable resources while also delivering major benefits to the energy storage field. Some biopolymer-derived porous materials exhibit interconnected porous architecture, as well as heteroatom doping, physicochemical stability, and various nanostructures, resulting in improved performance rate, high capacitance, and cyclic stability. Furthermore, the formation of composite materials combining biopolymer-derived materials with conductive materials, such as graphene, transition metals, and conductive polymers, results in efficient charge transfer, rapid redox reaction kinetics, and durable structures, resulting in hybrids with a long-life cycle and high energy density. The synthesis methods and the obtained structure will inform the application of the various biopolymer-based materials for various applications, as highlighted in Figure 18. Although a great number of experts have put in a lot of effort to make biopolymer-based materials suitable for use in supercapacitors, there are still a few obstacles that will need to be overcome soon.

(1) Scalable synthesis: green biopolymers come from a variety of sources; nevertheless, their production is complex, expensive, and challenging on a large scale, requiring the separation of certain components from the green biopolymeric resources and the incorporation of other electrochemically active materials. Because of the different sources of biopolymers, the flexibility and mechanical strength of certain derived materials are insufficient for practical use.
(2) Advanced characterization and evaluation of supercapacitor biopolymer-derived electrode materials are extremely promising as materials for various components. In situ infrared spectroscopy, in situ TEM, AFM, in situ SEM, and in situ XRD should be used to further investigate their synthesis and interfacial reaction processes. Furthermore, by integrating further experiments with theoretical calculations, a deeper understanding of the electrochemical processes, structural and textural properties of the synthesized materials can be obtained. This would provide intriguing directions for future research.
(3) Control of structure–properties–performance: Biopolymers, as a type of natural material, have unique properties that do not quite meet the size necessary for ionic mobility in supercapacitors, hence managing these naturally generated pores remains a difficulty. At the moment, chemical approaches for changing the pore size of biopolymers are effective. The electrochemical performance of the electrode made from the modified biopolymer has improved, but it still falls short of the requirements. Based on this, electrode materials with uniform pore size and specific surface area distribution may be generated using proper design and synthesis procedures, allowing high performance supercapacitors to be assembled. With the continuous research and exploration of green biopolymer-based materials, we surely believe that these renewable carbon resources will replace traditional petroleum-based products and obtain extensive applications in the energy storage field.

## Figures and Tables

**Table 1:** Overview of electrode materials synthesized from biopolymer-based materials for supercapacitor applications.

Biopolymer	Synthesis Method	Material Obtained	SSA (m^2^/g)	C_s_ (F/g)	Retention (%)	Ref
Lignin	Electrospinning; Heat treatment	2-D carbon nanofiber	807	203.29 @ 1 A/g	99.6 (10,000 cycles)	[38]
Lignin	Electrospinning; Heat treatment	Carbon nanofibers	1363	289 @ 0.1 A/g	92 (10,000 cycles)	[39]
Alginate	In-situ hard template	Porous carbon	1838	350 @ 1 A/g	90.4 (5000 cycles)	[34]
Cellulose	Crosslinking sol-gel method	Co-doped porous carbon	1501	358 @ 1 A/g	97.8 (10,000 cycles)	[31]
Agar	Carbonization-activation	Co-doped activated carbon	2134	313.7 @ 0.5 A/g	~100 (50,000 cycles)	[20]
Chitosan	Microwave-assisted method	N-doped porous carbon	1170	435 @ 0.2 A/g	~100 (cycles)	[33]
Chitosan	Hydrothermal	Chitosan/rGO composite	67	274 @ 0.5 A/g	92.57 (10,000 cycles)	[8]
Alginate	Microwave-assisted method	Nanospheres	-	1329 @ 2 mA	91 (1000 cycles)	[11]
Pectin	In-situ template	3D porous carbon	2928	338 @ 1 A/g	85 (5000 cycles)	[40]
Sodium lignosulfonate	Self-template	Porous carbon	529	170 @ 0.5 A/g	81 (5000 cycles)	[41]
Xanthan gum and soy protein	Dual-template	N-doped carbon aerogel	1438	264.3 @ 0.5 A/g	98.6 (10,000 cycles)	[42]
Human hair	Microwave-assisted method	Reduced graphene oxide	-	316 @ 1 A/g	89 (3000 cycles)	[43]
Chitosan	Hydrothermal	Co-doped porous carbon	3646	586 @ 1 A/g	97 (10,000 cycles)	[44]
Lignin	Hydrothermal carbonization and activation	Porous carbon	1660	218 @ 1 A/g	99 (10,000 cycles)	[45]
Lignin	Spray drying; KOH activation	Porous carbon	1590	340 @ 0.5 A/g	94 (5000 cycles)	[46]
Lignin	Spray drying; KOH activation	Hollow carbon sphere	2425	31.8 @ 0.2 A/g	-	[47]
Cellulose	Spray drying	Porous carbon	1096	194 @ 1 A/g	95.2 (5000 cycles)	[48]
Cellulose	Molten salt method	Carbon nanofiber	899	150 @ 1 A/g	96 (10,000 cycles)	[49]
Cellulose	Ice-templating carbonization	Porous carbon monoliths	724	210 @ 1 A/g	~100 (10,000 cycles)	[50]
Gelatin	Cross linking; activation	Porous carbon	1609	392 @ 1 A/g	83 (10,000 cycles)	[19]

**Table 2:** Electrochemical performance evaluation of some biopolymeric synthesized electrode materials.

Biopolymer Used	Fabrication Method	Derived Electrode Material	Current Density (A/g)	Specific Capacitance (F/g)	Energy Density (Wh/Kg)	Power Density (W/Kg)	Cycle Retention (%)	Ref.
**Collagen**	Feasible and simple approach	Mn-doped N-containing carbon	1	272.62	-	-	81.4	[105]
**Collagen**	Sol-gel, freeze-drying, carbonization, and KOH activation	Nitrogen-doped carbon aerogels	0.5	185.3	-	-	90.2	[106]
**Chitin**	New facile method	Heteroatom-doped hierarchical porous carbon (HPC-700)	0.5	412.5	9.67	-	96.6	[93]
**Chitin**	Sol-gel transition in NaOH/Urea solvent, followed by carbonization	Chitin nanoparticles yielding activated carbon (ACNC-800)	0.5	245	-	-	98	[95]
**Marine crustacean derivatives/chitin nanofibers (ChNF)**	Bio-templates of zeolitic imidazolate frameworks (ZIF-8), followed by carbonization	Biobased N-doped hierarchically porous carbon (N-HPC-900)	0.2	182.5	4.46	50	94.79	[94]
**Chitin**	Hydroxyapatite template (HAP)	N-doped carbon material (NC-HAP-700)	0.5	346	-	-	92	[107]
**Chitin**	Pre-carbonization in air, followed by KOH activation	Cicada slough-derived carbon (CSC-2)	0.5	266.5	15.97	5000	92.7	[108]
**Starch**	Two-step procedure	Ultrathin carbon nanosheet-supported Ni quantum dot hybrids (C-Ni-QDs)	2	1120	-	-	97	[86]
**Starch**	TiO2 activated starch template	A-TiO2	-	388	194	4473	99	[87]
**Starch**	Gelatinization of the starch using EtOH/H2O	Gelatinized starch-derived activated carbon (SAC)	0.5	335.6	-	-	96	[109]
**Starch**	Sol-gel technique	Binder-free activated carbon	1	272	18–25		75.9	[110]
**Potato starch**	Hydrothermal method and high temperature carbonization	Uniform and monodisperse carbon microspheres (MCMSs)	1	245	759		61	[111]
**Corn starch**	Hydrothermal carbonization and chemical activation with H3PO4	Porous carbon	0.625	144	19.9	311	99	[84]
**Chitosan**	Electrostatic contact	Sandwich-like structure (CPCM/MXene)	0.5	362	-	-	93.87	[88]
**Chitosan**	New phytic acid-induced self-assembled chitosan aerogel synthesis, followed by pyrolysis/activation	N, P-co-doped porous carbon materials (NPPCs)	1	231.2	-	-	96.7	[90]
**Chitosan**	Covering graphene slurry with weight percentages (including 5, 10, 15, 20, and 25% wt percent) of chitosan	10 %-graphene–chitosan electrodes	0.4	114.284	80.40	2894.6	87.2	[112]
**Chitosan**	One-step carbonization technique	Nitrogen and sulphur co-doped porous chitosan hydrogel-derived carbons (CHC-SK)	0.5	366.8	-	-	97.1	[89]
**Chitosan**	Green synthesis approach	Ag-Zirconia nanocomposite	1	256	31.94	500.86	89	[92]

## Data Availability

Not applicable.

## References

[1] Vasudevan L.S.A.M., Perumal V., Ovinis M., Raja P.B., Edison T.N.J.I. (2021). Bioresource-derived polymer composites for energy storage applications: Brief review. J. Environ. Chem. Eng..

[2] Gao Z., Zhang Y., Song N., Li X. (2016). Biomass-derived renewable carbon materials for electrochemical energy storage. Mater. Res. Lett..

[3] Ranaweera C.K., Kahol P.K., Ghimire M., Mishra S.R., Gupta R.K. (2017). Orange-Peel-Derived Carbon: Designing Sustainable and High-Performance Supercapacitor Electrodes. C.

[4] Kim B.K., Sy S., Yu A., Zhang J. (2015). Electrochemical Supercapacitors for Energy Storage and Conversion. Handbook of Clean Energy Systems.

[5] Mensah-Darkwa K., Zequine C., Kahol P.K., Gupta R.K. (2019). Supercapacitor energy storage device using biowastes: A sustainable approach to green energy. Sustainability.

[6] Loganathan N.N., Perumal V., Pandian B.R., Atchudan R., Edison T.N.J.I., Ovinis M. (2022). Recent studies on polymeric materials for supercapacitor development. J. Energy Storage.

[7] Yang Z., Ren J., Zhang Z., Chen X., Guan G., Qiu L., Zhang Y., Peng H. (2015). Recent Advancement of Nanostructured Carbon for Energy Applications. Chem. Rev..

[8] Gao M., Wang L., Zhao B., Gu X., Li T., Huang L., Wu Q., Yu S., Liu S. (2021). Sandwich construction of chitosan/reduced graphene oxide composite as additive-free electrode material for high-performance supercapacitors. Carbohydr. Polym..

[9] Sun J., Liu Z., Rujiralai T., Chen J., Ma L., Chen H., Liu Y., Liao L., Chen K., Xi Y. (2022). Tungsten disulfide nanoparticles embedded in gelatin-derived honeycomb-like nitrogen-doped carbon networks with reinforced electrochemical pseudo-capacitance performance. J. Energy Storage.

[10] Sekretaryova A. (2020). Powering wearable bioelectronic devices. Wearable Bioelectronics.

[11] Jeyabanu K., Devendran P., Manikandan A., Packiaraj R., Ramesh K., Nallamuthu N. (2019). Preparation and characterization studies of La doped CuS nanospheres by microwave irradiation for high performance supercapacitors. Phys. B Condens. Matter.

[12] Li Y., Zhang D., He J., Wang Y., Zhang X., Zhang Y., Liu X., Wang K., Wang Y. (2019). Hierarchical porous carbon nanosheet derived from waste engine oil for high-performance supercapacitor application. Sustain. Energ. Fuels.

[13] Sun Z., Qu K., You Y., Huang Z., Liu S., Li J., Hu Q., Guo Z. (2021). Overview of cellulose-based flexible materials for supercapacitors. J. Mater. Chem. A.

[14] Bragulla H.H., Homberger D.G. (2009). Structure and functions of keratin proteins in simple, stratified, keratinized and cornified epithelia. J. Anat..

[15] Angel N., Li S., Yan F., Kong L. (2022). Recent advances in electrospinning of nanofibers from bio-based carbohydrate polymers and their applications. Trends Food Sci. Technol..

[16] Kebabsa L., Kim J., Lee D., Lee B. (2020). Highly porous cobalt oxide-decorated carbon nanofibers fabricated from starch as free-standing electrodes for supercapacitors. Appl. Surf. Sci..

[17] Chen H., Yu F., Wang G., Chen L., Dai B., Peng S. (2018). Nitrogen and Sulfur Self-Doped Activated Carbon Directly Derived from Elm Flower for High-Performance Supercapacitors. ACS Omega.

[18] Peng K., Wang W., Zhang J., Ma Y., Lin L., Gan Q., Chen Y. (2022). Preparation of chitosan/sodium alginate conductive hydrogels with high salt contents and their application in flexible supercapacitors. Carbohydr. Polym..

[19] Wang G., Lin Z., Jin S., Li M., Jing L. (2022). Gelatin-derived honeycomb like porous carbon for high mass loading supercapacitors. J. Energy Storage.

[20] Guo Y., Wang T., Chen X., Wu D. (2021). Agar-based porous electrode and electrolyte for flexible symmetric supercapacitors with ultrahigh energy density. J. Power Sources.

[21] Ababneh H., Hameed B.H. (2021). Chitosan-derived hydrothermally carbonized materials and its applications: A review of recent literature. Int. J. Biol. Macromol..

[22] Shu Y., Bai Q., Fu G., Xiong Q., Li C., Ding H. (2020). Hierarchical porous carbons from polysaccharides carboxymethyl cellulose, bacterial cellulose, and citric acid for supercapacitor. Carbohydr. Polym..

[23] Wang X., Xie X., Xie K., Liu Q., Li Y., Tan X., Dong H., Li X., Dong Z., Xia Q. (2022). Chitin and cuticle proteins form the cuticular layer in the spinning duct of silkworm. Acta Biomater..

[24] Liu Y., Chen J., Cui B., Yin P., Zhang C. (2018). Design and Preparation of Biomass-Derived Carbon Materials for Supercapacitors: A Review. C.

[25] Yang X., Fei B., Ma J., Liu X., Yang S., Tian G., Jiang Z. (2018). Porous nanoplatelets wrapped carbon aerogels by pyrolysis of regenerated bamboo cellulose aerogels as supercapacitor electrodes. Carbohydr. Polym..

[26] Zhuo H., Hu Y., Chen Z., Zhong L. (2019). Cellulose carbon aerogel/PPy composites for high-performance supercapacitor. Carbohydr. Polym..

[27] Joshi B., Samuel E., Kim Y.-I., Yarin A.L., Swihart M.T., Yoon S.S. (2022). Review of recent progress in electrospinning-derived freestanding and binder-free electrodes for supercapacitors. Coord. Chem. Rev..

[28] Butnoi P., Pangon A., Berger R., Butt H.-J., Intasanta V. (2021). Electrospun nanocomposite fibers from lignin and iron oxide as supercapacitor material. J. Mater. Res. Technol..

[29] Wang Y., Qu Q., Cui J., Lu T., Li F., Zhang M., Liu K., Zhang Q., He S., Huang C. (2021). Design and fabrication of cellulose derived free-standing carbon nanofiber membranes for high performance supercapacitors. Carbohydr. Polym. Technol. Appl..

[30] Fan Q., Ma C., Wu L., Wei C., Wang H., Song Y., Shi J. (2019). Preparation of cellulose acetate derived carbon nanofibers by ZnCl_2_ activation as a supercapacitor electrode. RSC Adv..

[31] Lin S., Mo L., Wang F., Shao Z. (2021). N/O co-doped hierarchically porous carbon with three-dimensional conductive network for high-performance supercapacitors. J. Alloys Compd..

[32] Ren R., Zhong Y., Ren X., Fan Y. (2022). Chitosan-based oxygen-doped activated carbon/graphene composite for flexible supercapacitors. RSC Adv..

[33] Li B., Cheng Y., Dong L., Wang Y., Chen J., Huang C., Wei D., Feng Y., Jia D., Zhou Y. (2017). Nitrogen doped and hierarchically porous carbons derived from chitosan hydrogel via rapid microwave carbonization for high-performance supercapacitors. Carbon.

[34] Zhou Y., Ren X., Song M., Du Y., Wan J., Wu G., Ma F. (2020). In-situ template cooperated with thiourea to prepare oxygen/nitrogen co-doped porous carbons with adjustable pore structure for supercapacitors. Renew. Energy.

[35] Zhang Q., Liu D., Pei H., Pan W., Liu Y., Xu S., Cao S. (2022). Swelling-reconstructed chitosan-viscose nonwoven fabric for high-performance quasi-solid-state supercapacitors. J. Colloid Interface Sci..

[36] Li K., Li P., Sun Z., Shi J., Huang M., Chen J., Liu S., Shi Z., Wang H. (2022). All-cellulose-based quasi-solid-state supercapacitor with nitrogen and boron dual-doped carbon electrodes exhibiting high energy density and excellent cyclic stability. Green Energy Environ..

[37] Song Y., Liu J., Sun K., Xu W. (2017). Synthesis of sustainable lignin-derived mesoporous carbon for supercapacitors using a nano-sized MgO template coupled with Pluronic F127. RSC Adv..

[38] Singh M., Gupta A., Sundriyal S., Dubey P., Jain K., Dhakate S. (2022). Activated green carbon-based 2-D nanofabric mats for ultra-flexible all-solid-state supercapacitor. J. Energy Storage.

[39] Ma C., Wu L., Dirican M., Cheng H., Li J., Song Y., Shi J., Zhang X. (2021). ZnO-assisted synthesis of lignin-based ultra-fine microporous carbon nanofibers for supercapacitors. J. Colloid Interface Sci..

[40] Zhou Y., Ren X., Du Y., Jiang Y., Wan J., Ma F. (2020). In-situ template cooperated with urea to construct pectin-derived hierarchical porous carbon with optimized pore structure for supercapacitor. Electrochim. Acta.

[41] Zhang X., Jian W., Zhao L., Wen F., Chen J., Yin J., Qin Y., Lu K., Zhang W., Qiu X. (2021). Direct carbonization of sodium lignosulfonate through self-template strategies for the synthesis of porous carbons toward supercapacitor applications. Colloids Surf. A Physicochem. Eng. Asp..

[42] Li P., Xie H., Liu Y., Wang J., Wang X., Xie Y., Hu W., Xie T., Wang Y., Zhang Y. (2020). Dual-templated 3D nitrogen-enriched hierarchical porous carbon aerogels with interconnected carbon nanosheets from self-assembly natural biopolymer gel for supercapacitors. Electrochim. Acta.

[43] Zhao J., Gong J., Zhou C., Miao C., Hu R., Zhu K., Cheng K., Ye K., Yan J., Cao D. (2020). Utilizing human hair for solid-state flexible fiber-based asymmetric supercapacitors. Appl. Surf. Sci..

[44] Xin X., Song N., Jia R., Wang B., Dong H., Ma S., Sui L., Chen Y., Zhang Q., Dong L. (2021). N, P-codoped porous carbon derived from chitosan with hierarchical N-enriched structure and ultra-high specific surface Area toward high-performance supercapacitors. J. Mater. Sci. Technol..

[45] Guo N., Li M., Sun X., Wang F., Yang R. (2017). Enzymatic hydrolysis lignin derived hierarchical porous carbon for supercapacitors in ionic liquids with high power and energy densities. Green Chem..

[46] Tomaszewska A., Chu Z., Feng X., O’Kane S., Liu X., Chen J., Ji C., Endler E., Li R., Liu L. (2019). Lithium-ion battery fast charging: A review. eTransportation.

[47] Cao K.L.A., Kitamoto Y., Iskandar F., Ogi T. (2021). Sustainable porous hollow carbon spheres with high specific surface area derived from Kraft lignin. Adv. Powder Technol..

[48] Wang C., Wang X., Lu H., Li H., Zhao X. (2018). Cellulose-derived hierarchical porous carbon for high-performance flexible supercapacitors. Carbon.

[49] Zhong Y., Wang T., Yan M., Huang X., Zhou X. (2022). Carbon nanofibers derived from cellulose via molten-salt method as supercapacitor electrode. Int. J. Biol. Macromol..

[50] Lee B.M., Jeong C.U., Hong S.K., Yun J.M., Choi J.H. (2020). Eco-friendly fabrication of porous carbon monoliths from water-soluble carboxymethyl cellulose for supercapacitor applications. J. Ind. Eng. Chem..

[51] Sinha P., Yadav A., Tyagi A., Paik P., Yokoi H., Naskar A.K., Kuila T., Kar K.K. (2020). Keratin-derived functional carbon with superior charge storage and transport for high-performance supercapacitors. Carbon.

[52] Wu S., Zhou H., Zhou Y., Wang H., Li Y., Liu X., Zhou Y. (2021). Keratin-derived heteroatoms-doped hierarchical porous carbon materials for all-solid flexible supercapacitors. J. Alloys Compd..

[53] Chen H., Lei X., Yu T., Guan X., Yuan H. (2021). Ultra-high specific capacitance of self-doped 3D hierarchical porous turtle shell-derived activated carbon for high-performance supercapacitors. Ceram. Int..

[54] Liu W., Feng K., Zhang Y., Yu T., Han L., Lui G., Li M., Chiu G., Fung P., Yu A. (2017). Hair-based flexible knittable supercapacitor with wide operating voltage and ultra-high rate capability. Nano Energy.

[55] Dev K., Sinha P., Ghorai M.K., Kar K.K. (2022). Mesoporous electrode from human hair and bio-based gel polymer electrolyte for high-performance supercapacitor. Diam. Relat. Mater..

[56] Zhang L., Meng Z., Qi Q., Yan W., Lin N., Liu X.Y. (2018). Aqueous supercapacitors based on carbonized silk electrodes. RSC Adv..

[57] Wang B., Li D., Tang M., Ma H., Gui Y., Tian X., Quan F., Song X., Xia Y. (2018). Alginate-based hierarchical porous carbon aerogel for high-performance supercapacitors. J. Alloys Compd..

[58] Sun S., Ding B., Liu R., Wu X. (2019). Facile synthesis of three-dimensional interconnected porous carbon derived from potassium alginate for high performance supercapacitor. J. Alloys Compd..

[59] Xia L., Huang H., Fan Z., Hu D., Zhang D., Sammed A., Usman M., Pan L. (2019). Hierarchical macro-/meso-/microporous oxygen-doped carbon derived from sodium alginate: A cost-effective biomass material for binder-free supercapacitors. Mater. Des..

[60] Wei K., Zhang F., Yang Y., Zhai B., Wang X., Song Y. (2022). Oxygenated N-doped porous carbon derived from ammonium alginate: Facile synthesis and superior electrochemical performance for supercapacitor. J. Energy Storage.

[61] Li Y., Liu X., Gong Q., Xia Z., Yang Y., Chen C., Qian C. (2021). Facile preparation of stretchable and self-healable conductive hydrogels based on sodium alginate/polypyrrole nano fibers for use in flexible supercapacitor and strain sensors. Int. J. Biol. Macromol..

[62] Zhao Y., Wei M., Zhu Z., Zhang J., Xiao L., Hou L. (2019). Facile preparation of N-O codoped hierarchically porous carbon from alginate particles for high performance supercapacitor. J. Colloid Interface Sci..

[63] Um J., Manguy J., Anes J., Jacquier J.C., Hurley D., Dillon E.T., Wynne K., Fanning S., O’Sullivan M., Shields D.C. (2021). Enriching antimicrobial peptides from milk hydrolysates using pectin/alginate food-gels. Food Chem..

[64] Waatriah E., Shahrin E.S., Alimatul N., Narudin H., Maharani K., Kusrini E., Hanif A., Ningsheh N., Shahri M., Usman A. (2021). Pectin derived from pomelo pith as a superior adsorbent to remove toxic Acid Blue 25 from aqueous solution. Carbohydr. Polym. Technol. Appl..

[65] Wang C., Li G., Karmakar B., Al Salem H.S., El-kott A.F., Elsaid F.G., Bani-Fwaz M.Z., Alsayegh A.A., Alkhayyat S.S., Batiha G.E. (2022). Pectin mediated green synthesis of Fe3O4/Pectin nanoparticles under ultrasound condition as an anti-human colorectal carcinoma bionanocomposite. Arab. J. Chem..

[66] Perumal P., Selvin P.C. (2021). Boosting the performance of electric double layer capacitor via engaging pectin macromolecular electrolyte with elevated ionic conductivity and potential window stability. Chem. Eng. J. Adv..

[67] Bakhtiarian M., Mehdi M. (2021). Sonochemical synthesis of 1, 4-dihydropyridines using a bio-derived magnetic nanocomposite based on the pectin modified with the disulfonic acids under mild conditions. Mater. Today Commun..

[68] Xu L., Cui L., Jia M., Li Y., Gao J., Jin X. (2018). Self-assembly of flexible graphene hydrogel electrode based on crosslinked. Carbohydr. Polym..

[69] Jie Z., Ying X. (2018). Illustrating the effect of electron withdrawing and electron donating groups adherent to p-hydroquinone on supercapacitor performance: The cases of sulfonic acid and methoxyl groups. Electrochim. Acta.

[70] Stanisz M., Klapiszewski Ł., Jesionowski T. (2020). Recent advances in the fabrication and application of biopolymer-based micro- and nanostructures: A comprehensive review. Chem. Eng. J..

[71] Saliu O.D., Mamo M., Ndungu P., Ramontja J. (2021). The making of a high performance supercapacitor active at negative potential using sulphonic acid activated starch-gelatin-TiO2 nano-hybrids. Arab. J. Chem..

[72] Wu C.-W., Li P.-H., Wei Y.-M., Yang C., Wu W.-J. (2022). Review on the preparation and application of lignin-based carbon aerogels. RSC Adv..

[73] Jiang G., Senthil R.A., Sun Y., Kumar T.R., Pan J. (2021). Recent progress on porous carbon and its derivatives from plants as advanced electrode materials for supercapacitors. J. Power Sources.

[74] Jha S., Mehta S., Chen Y., Ma L., Renner P., Parkinson D.Y., Liang H. (2020). Design and Synthesis of Lignin-Based Flexible Supercapacitors. ACS Sustain. Chem. Eng..

[75] Wang H., Fu F., Huang M., Feng Y., Han D., Xi Y., Xiong W., Yang D., Niu L. (2022). Lignin-based materials for electrochemical energy storage devices. Nano Mater. Sci..

[76] Schlee P., Hosseinaei O., Baker D., Landmér A., Tomani P., Mostazo-López M.J., Cazorla-Amorós D., Herou S., Titirici M.-M. (2019). From Waste to Wealth: From Kraft Lignin to Free-standing Supercapacitors. Carbon.

[77] Schlee P., Herou S., Jervis R., Shearing P.R., Brett D.J.L., Baker D., Hosseinaei O., Tomani P., Murshed M.M., Li Y. (2019). Free-standing supercapacitors from Kraft lignin nano-fibers with remarkable volumetric energy density. Chem. Sci..

[78] Selvaraj T., Perumal V., Khor S.F., Anthony L.S., Gopinath S.C.B., Mohamed N.M. (2020). The recent development of polysaccharides biomaterials and their performance for supercapacitor applications. Mater. Res. Bull..

[79] Wang C., Wu D., Wang H., Gao Z., Xu F., Jiang K. (2018). A green and scalable route to yield porous carbon sheets from biomass for supercapacitors with high capacity. J. Mater. Chem. A.

[80] Hao X., Wang J., Ding B., Wang Y., Chang Z., Dou H., Zhang X. (2017). Bacterial-cellulose-derived interconnected meso-microporous carbon nanofiber networks as binder-free electrodes for high-performance supercapacitors. J. Power Sources.

[81] Mo R., Zhao Y., Zhao M., Wu M., Wang C., Li J. (2018). Graphene-like porous carbon from sheet cellulose as electrodes for supercapacitors. Chem. Eng. J..

[82] Luo H., Liu J., Chen Y., Xie Z. (2021). Microcrystalline cellulose derived hierarchically porous nanocarbons via a template-free method for high performance supercapacitors. Diam. Relat. Mater..

[83] Kasturi P.R., Ramasamy H., Meyrick D., Lee Y.S., Selvan R.K. (2019). Preparation of starch-based porous carbon electrode and biopolymer electrolyte for all solid-state electric double layer capacitor. J. Colloid Interface Sci..

[84] Pang L., Zou B., Zou Y., Han X., Cao L., Wang W., Guo Y. (2016). Aspects A new route for the fabrication of corn starch-based porous carbon as electrochemical supercapacitor electrode material. Colloids Surf. A Physicochem. Eng. Asp..

[85] Li Z., Liu Q., Sun L., Li N., Wang X., Wang Q., Zhang D., Wang B. (2021). Nitrogen and oxygen Co-doped porous carbon derived from yam waste for high-performance supercapacitors. RSC Adv..

[86] Chen T., Liu Z. (2019). Starch-assistant synthesis of Ni quantum dots/ultrathin carbon nanosheet hybrids for high performance supercapacitor. Mater. Lett..

[87] Saliu O., Mamo M., Ndungu P., Ramontja J. (2022). Starch built TiO2 nanoarchitecture with mixed anatase and rutile phase for high energy density supercapacitor electrode. J. Energy Storage.

[88] Wei L., Deng W., Li S., Wu Z., Cai J., Luo J. (2022). Sandwich-like chitosan porous carbon Spheres/MXene composite with high specific capacitance and rate performance for supercapacitors. J. Bioresour. Bioprod..

[89] Lv S., Ma L., Shen X., Tong H. (2022). Nitrogen and sulfur co-doped porous chitosan hydrogel-derived carbons for supercapacitors. J. Electroanal. Chem..

[90] Xiao J., Wang Y., Zhang T.C., Ouyang L., Yuan S. (2022). Phytic acid-induced self-assembled chitosan gel-derived N, P–co-doped porous carbon for high-performance CO 2 capture and supercapacitor. J. Power Sources.

[91] Ba Y., Pan W., Pi S., Mi L. (2018). Nitrogen-doped hierarchical porous carbon derived from a chitosan/polyethylene glycol blend for high performance supercapacitors. RSC Adv..

[92] Nayak S., Kittur A.A., Nayak S. (2022). Green synthesis of Silver-Zirconia composite using chitosan biopolymer binder for fabrication of electrode materials in supercapattery application for sustainable energy storage. Curr. Res. Green Sustain. Chem..

[93] Wang Y., Liu R., Tian Y., Sun Z., Huang Z., Wu X., Li B. (2019). Heteroatoms-doped hierarchical porous carbon derived from chitin for flexible all-solid-state symmetric supercapacitors. Chem. Eng. J..

[94] Shang Z., An X., Liu L., Yang J., Zhang W., Dai H., Cao H., Xu Q., Liu H., Ni Y. (2020). Chitin nanofibers as versatile bio-templates of zeolitic imidazolate frameworks for N-doped hierarchically porous carbon electrodes for supercapacitor. Carbohydr. Polym..

[95] Zheng S., Zhang J., Deng H., Du Y., Shi X. (2021). Chitin derived nitrogen-doped porous carbons with ultrahigh specific surface area and tailored hierarchical porosity for high performance supercapacitors. J. Bioresour. Bioprod..

[96] Zheng S., Cui Y., Zhang J., Gu Y., Shi X., Peng C., Wang D. (2019). Nitrogen doped microporous carbon nanospheres derived from chitin nanogels as attractive materials for supercapacitors. RSC Adv..

[97] Antonio J.D.S., Jacenko O., Fertala A., Orgel J.P.R.O. (2021). Collagen structure-function mapping informs applications for regenerative medicine. Bioengineering.

[98] Söder S., Hambach L., Lissner R., Kirchner T., Aigner T. (2002). Ultrastructural localization of type VI collagen in normal adult and osteoarthritic human articular cartilage. Osteoarthr. Cartil..

[99] Chandrasekaran P., Kwok B., Han B., Adams S.M., Wang C., Chery D.R., Mauck R.L., Dyment N.A., Lu X.L., Frank D.B. (2021). Type V collagen regulates the structure and biomechanics of TMJ condylar cartilage: A fibrous-hyaline hybrid. Matrix Biol..

[100] Wu Z., Korntner S., Mullen A., Zeugolis D. (2021). Collagen type II: From biosynthesis to advanced biomaterials for cartilage engineering. Biomater. Biosyst..

[101] Gelse K., Pöschl E., Aigner T. (2003). Collagens—Structure, function, and biosynthesis. Adv. Drug Deliv. Rev..

[102] Owczarzy A., Kurasiński R., Kulig K., Rogóż W., Szkudlarek A., Maciążek-Jurczyk M. (2020). Collagen-structure, properties and application. Eng. Biomater..

[103] Kennedy L.J., Ratnaji T., Konikkara N., Vijaya J.J. (2018). Value added porous carbon from leather wastes as potential supercapacitor electrode using neutral electrolyte. J. Clean. Prod..

[104] Shaali R., Doroodmand M.M., Moazeni M. (2021). Supercapacitance/Resistance Behaviors of Helminth Eggs as Reliable Recognition and Direct Differentiation Probe. Front. Bioeng. Biotechnol..

[105] Lei J., Zhou J., Li J., Wen J., Su L., Duan T., Zhu W. (2018). Novel collagen waste derived Mn-doped nitrogen-containing carbon for supercapacitors. Electrochim. Acta.

[106] Yu M., Han Y., Li J., Wang L. (2018). Magnetic N-doped carbon aerogel from sodium carboxymethyl cellulose/collagen composite aerogel for dye adsorption and electrochemical supercapacitor. Int. J. Biol. Macromol..

[107] Chu M., Zhai Y., Shang N., Guo P., Wang C., Gao Y. (2020). N-doped carbon derived from the monomer of chitin for high-performance supercapacitor. Appl. Surf. Sci..

[108] Zhou J., Bao L., Wu S., Yang W., Wang H. (2017). Chitin based heteroatom-doped porous carbon as electrode materials for supercapacitors. Carbohydr. Polym..

[109] Zhao C., Wang Y., Zheng J., Xu S., Rui P., Zhao C. (2021). Improved supercapacitor performance of α-starch-derived porous carbon through gelatinization. J. Power Sources.

[110] Zhong Y., Shi T., Huang Y., Cheng S., Liao G., Tang Z. (2018). One-step synthesis of porous carbon derived from starch for all-carbon binder-free high-rate supercapacitor. Electrochim. Acta.

[111] Ruibin Q., Zhongai H., Yuying Y., Zhimin L., Ning A., Xiaoying R., Haixiong H., Hongying W. (2015). Monodisperse carbon microspheres derived from potato starch for asymmetric supercapacitors. Electrochim. Acta.

[112] Azmira N., Kheawhom S., Azmin A. (2021). Chitosan as biopolymer binder for graphene in supercapacitor electrode. Results Phys..

